# Ectopic mouse TMC1 and TMC2 alone form mechanosensitive channels that are potently modulated by TMIE

**DOI:** 10.1073/pnas.2403141122

**Published:** 2025-02-25

**Authors:** Yixuan Chen, Yulin Li, Yonghong Liu, Jiawen Sun, Wanying Feng, Yanfei Chen, Ye Tian, Tianlun Lei, Pingbo Huang

**Affiliations:** ^a^Department of Chemical and Biological Engineering, Hong Kong University of Science and Technology, Hong Kong 0000, China; ^b^Division of Life Science, Hong Kong University of Science and Technology, Hong Kong 0000, China; ^c^State Key Laboratory of Molecular Neuroscience, Hong Kong University of Science and Technology, Hong Kong 0000, China

**Keywords:** TMC1/2, MT channel, cell-surface expression, Fyn lipidation tag, TMIE

## Abstract

The mechanotransduction (MT) channel expressed in cochlear and vestibular hair cells converts the mechanical stimulation of sound and head movements into electrochemical signals. Recently, TMC1 and TMC2 (TMC1/2) have been recognized as the pore-forming subunit of the MT channel, but TMC1/2 functional expression in heterologous cells—which is critical for unequivocally identifying them as the bona fide pore-forming subunit of the MT channel—has not been achieved because ectopic TMC1/2 become trapped in the ER. Here, we report that adding a Fyn lipidation tag to mouse TMC1/2 (mTMC1/2) drove their cell-surface expression, and, importantly, full-length mTMC1/2 expressed alone functioned as mechanosensitive channels, underscoring the view that TMC1/2 constitute the sole pore-forming subunit of the MT channel. Moreover, mouse transmembrane inner ear (TMIE) (mTMIE) protein robustly stimulated TMC1/2 channel activity by modulating their gating. Intriguingly, the N-terminal 27 residues of mTMIE were dispensable for regulating TMC1/2 in our in vitro functional assay, whereas, in striking contrast, mutating mTMIE C76C77, the predicted palmitoylation sites, eliminated mTMIE stimulation of mTMC1/2, indicating a crucial role of the palmitoyl group in regulating TMC1/2 gating. mTMC1/2+mTMIE form 18 pS and 24 pS single channels, respectively. mTMC1/2+mTMIE single channels showed biophysical and pharmacological properties similar to those of the MT channel. Our findings provide insights into several fundamental and debated aspects of the function of TMC1/2 and TMIE, and our functional assay of TMC1/2 and TMIE in heterologous cells will facilitate further functional and structural characterization of these proteins and other MT-complex components.

## Significance

Unraveling the molecular identity of the mechanotransduction (MT) channel has long been regarded as the “Holy Grail” in hearing research. Recently, TMC1 and TMC2 (TMC1/2) have emerged as the pore-forming subunit of the MT channel. However, the functional study of TMC1/2 in heterologous cells has been challenging due to their lack of plasmalemmal expression. Our findings represent a breakthrough to overcome this technical obstacle, shedding light on several fundamental questions regarding the function of TMC1/2 and transmembrane inner ear (TMIE) protein, another component of the MT channel. Moreover, these findings will facilitate further functional and structural studies of TMC1/2, TMIE, and other MT-complex components in vitro. Performing similar investigations in vivo would be time-consuming and complex, if not impossible.

Hair cells in the inner ear detect sound and head movements and convert these mechanical stimuli into electrochemical signals that can be processed by the central nervous system. This process is called mechanotransduction (MT), and the ion channel that mediates MT by inner-ear hair cells is called the MT channel [or mechanoelectrical transduction (MET) channel]. The MT channel has been intensively studied for four decades and its biophysical properties are well characterized, but the channel’s molecular identity remains incompletely characterized.

Recently, TMC1 and TMC2 (TMC1/2) have been recognized as the pore-forming subunit of the MT channel based on functional and structural studies (1–5), but the precise function of TMC1/2 has not been fully established. If a candidate protein is the bona fide MT channel in hair cells, ectopic expression of this protein must generate an ion channel that recapitulates the biophysical and pharmacological properties of the MT channel. However, ectopically expressed TMC1/2 become trapped in the ER and fail to reach the plasma membrane (6–8), which precludes electrophysiological characterization of TMC1/2 channel function. Consequently, TMC1/2 cell-surface delivery has been suggested to require the aid of TMC1 binding partners in either the MT complex or the trafficking pathway, but various combinations of these binding partners have failed to drive TMC1/2 cell-surface expression in studies conducted by various research groups (6, 8). Thus, cell-surface expression of ectopic TMC1/2 has emerged as a technical bottleneck in the unambiguous identification of TMC1/2 as the pore-forming subunit of the MT channel.

Recently, the aforementioned technical hurdle was circumvented by reconstituting in artificial liposomes truncated TMC1 from green sea turtle (CmTMC1) or TMC2 from budgerigar (MuTMC2) expressed in insect Sf9 cells; the truncated CmTMC1 or MuTMC2 in liposomes was reported to form a mechanosensitive (MS) channel (9). However, whether full-length mammalian TMC1/2 can form MS channels in heterologous cells is unknown. Adding yet another layer of complexity to the matter, TMC1/2 has been suggested to be incapable of forming MT channels in the absence of transmembrane inner ear (TMIE) protein, implying that TMIE is also part of the pore-forming components of the channel (10). This raises the question of whether mammalian TMC1 and TMC2 expressed alone, like CmTMC1 and MuTMC2, are sufficient for generating the MT channel. Considering these findings, we believe that it is of paramount importance to study the channel function of ectopic mammalian full-length TMC1/2, both in the absence and presence of TMIE. Here, we report that adding a Fyn lipidation tag to mouse TMC1/2 (mTMC1/2) promotes their cell-surface expression in heterologous cells, and, more importantly, that ectopic mTMC1 and mTMC2 expressed alone form MS channels in the absence of mouse TMIE (mTMIE), although their channel activity is strongly stimulated by mTMIE.

## Results

### Fyn Tag Drives Cell-Surface Expression of Ectopic Mouse and Human TMC1 and TMC2.

To investigate TMC1/2 channel function in heterologous cells, we used multiple approaches to drive their cell-surface expression in our initial experiments, but these efforts were unsuccessful. Lipidation target proteins to the membrane of distinct subcellular organelles and to the cell-surface membrane, and, importantly, lipidation also promotes the targeting of several ion channels to the cell surface (11). Therefore, we sought to test the effect of lipidation on the cell-surface expression of TMC1/2.

**Fig. 1.**
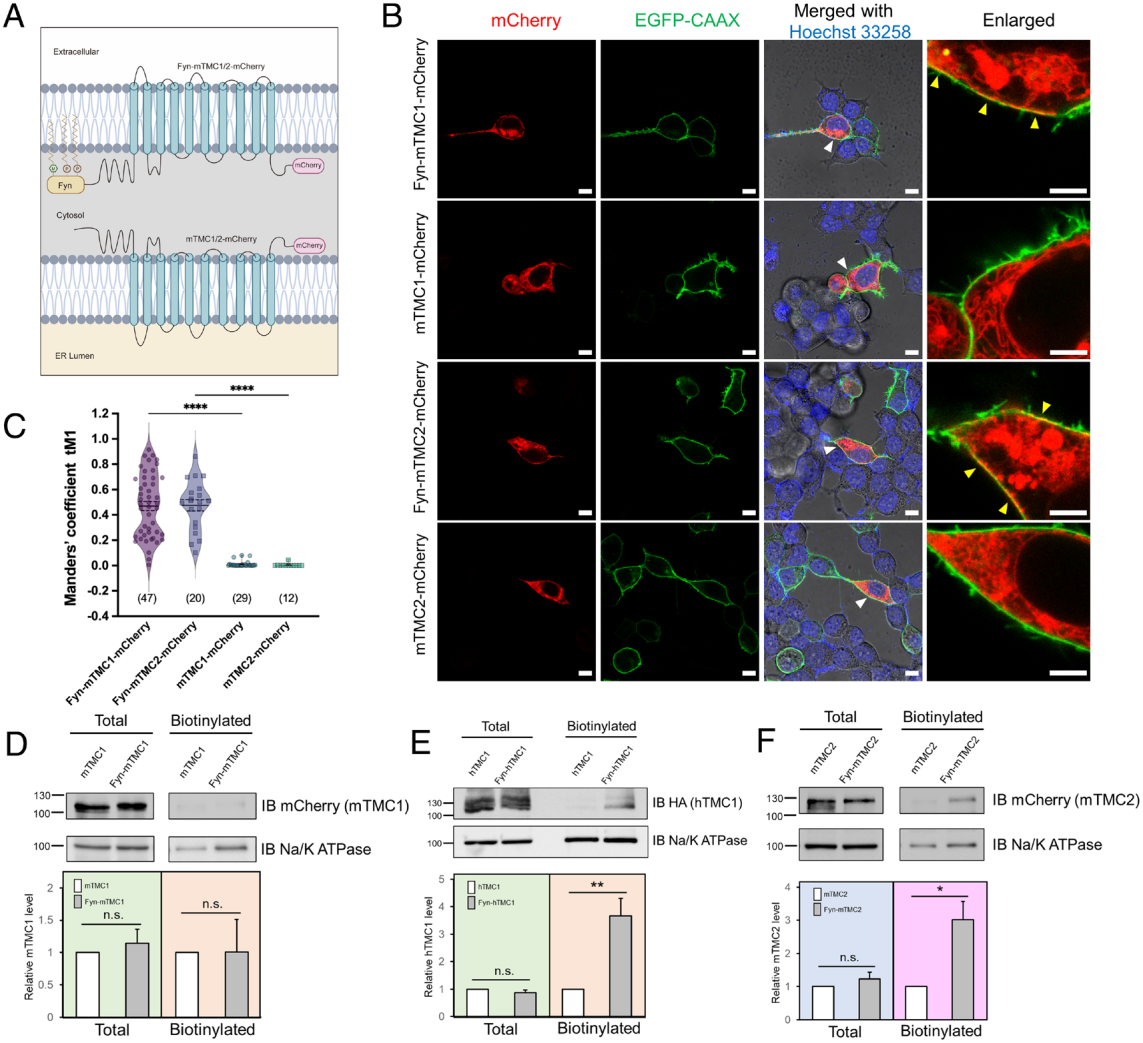
Fyn tag drives cell-surface expression of ectopic mouse TMC1/2 in PK-HEK293T cells. (*A*) Schematic of TMC1/2 with and without Fyn tag. Fyn-TMC1/2 and TMC1/2 localize in the plasma membrane (*Top*) and ER (*Bottom*), respectively. C-terminal mCherry tag facilitates visual identification of TMC1/2 in functional assays. (*B*) Confocal images of PK-HEK293T cells expressing mTMC1/2-mCherry (red) with or without Fyn tag. EGFP-CAAX (green): cell-surface membrane marker; Hoechst 33258 staining (blue): nuclei. Last column: enlargement of area indicated by the white arrowhead in the 3rd column; yellow arrowhead: overlap of Fyn-TMC1/2-mCherry with EGFP-CAAX. Scale bars: 10 μm, except for 5 μm in the last column. (*C*) Quantification of colocalization between Fyn-TMC1/2-mCherry (channel 1) and EGFP-CAAX (channel 2) using Manders’ coefficient. tM1 indicates the proportion of channel 2 signal that overlaps with channel 1 signal. Numbers in parentheses: number of cells tested; solid and dotted lines: mean and SEM, respectively. ****P < 0.0001. (*D*–*F*) Total (total) and cell-surface (biotinylated) levels of mTMC1-mCherry (*D*), hTMC1-tdTomato-HA (*E*), and mTMC2-mCherry (*F*) with or without Fyn tag. *Upper* panels: Representative western blots; *Lower* panels: summary data of four experiments similar to those in *Upper* panels. **P = 0.0059; *P = 0.011; n.s., not significant. Na/K ATPase: loading control. Very low amounts of TMC1/2 without Fyn tag were detected in “biotinylated” faction, presumably due to nonspecific binding to NeutrAvidin beads.

We engineered Fyn-TMC1 and Fyn-TMC2 by fusing at the N terminus of mTMC1/2 an 11-residue membrane-anchoring motif (MGCVQCKDKEA) derived from the Src kinase Fyn, which we named Fyn tag (Fig. 1*A*). This motif serves as a signal for myristoylation and palmitoylation, which aid stable anchoring of proteins to the plasma membrane and thereby promote their cell-surface expression (12). Notably, confocal microscopy analysis (Fig. 1*B* and *SI Appendix*, Fig. S4) revealed that Fyn-mTMC1/2-mCherry expressed in PIEZO1-knockout HEK293T (PK-HEK293T) cells reached the cell surface, as evidenced by their colocalization with the widely used plasmalemmal marker EGFP-CAAX, whereas mTMC1/2-mCherry (lacking the Fyn tag) became trapped in intracellular compartments. Fyn-mTMC1/2-mCherry exhibited an average colocalization of ~50% with plasmalemmal EGFP-CAAX, while mTMC1/2-mCherry showed only 1% or less colocalization with plasmalemmal EGFP-CAAX (Fig. 1*C*). The variability in colocalization of Fyn-mTMC1/2-mCherry with EGFP-CAAX may arise from differences in the lipidation of the Fyn tag and the plasmalemmal insertion of Fyn-mTMC1/2, which could be attributed to cell-to-cell heterogeneity (13). Furthermore, Fyn tag also targeted human TMC1 (hTMC1) to the plasma membrane of 3T3, COS7, and HEK293 cells (*SI Appendix*, Fig. S1), suggesting that the effect of the Fyn tag on TMC1/2 cell-surface expression is not species- or cell-type-specific.

Next, we assessed the plasmalemmal expression of Fyn-mTMC1/2-mCherry by using cell-surface biotinylation assays. Here, we detected only weak cell-surface biotinylation of Fyn-mTMC1-mCherry (Fig. 1*D*) despite its clear localization at the cell surface in confocal microscopy analysis (Fig. 1*B*); conversely, Fyn-hTMC1-tdTomato was cell-surface biotinylated effectively (Fig. 1*E*). We speculated that extracellular lysine residues in mTMC1 are buried inside the protein and thus inaccessible to biotinylation reagents; similar observations on other proteins have been reported (14). We utilized AlphaFold2 to predict the accessibility of extracellular lysines in mTMC1 and hTMC1(*SI Appendix*, Fig. S2). Interestingly, six out of seven extracellular lysines in both mTMC1 and hTMC1 are located very close to the membrane, making them inaccessible to Sulfo-NHS-SS-Biotin (MW, 606.7 Da)—the reagent used for cell-surface biotinylation. The only extracellular lysine that appears to be accessible is K482 in mTMC1 and K488 in hTMC1. However, the modeling also predicted that K482 and E486 in mTMC1 form a salt bridge, which is known to block acylation (15) and potentially other chemical modifications of lysine. By contrast, the residue equivalent to E486 of mTMC1 is an alanine (A492) in hTMC1. Therefore, our modeling analysis provides a plausible structural explanation for our experimental findings.

As an alternative, we used a plasma membrane protein isolation kit to examine the cell-surface expression of mTMC1: Agreeing with our microscopy results (Fig. 1*B*), the level of Fyn-mTMC1-mCherry in the plasma membrane fraction was significantly higher than that of mTMC1-mCherry (*SI Appendix*, Fig. S3). Importantly, Fyn-mTMC2-mCherry was also potently cell-surface biotinylated (Fig. 1*F*). We observed the presence of the biotinylated signal for hTMC1 (Fig. 1*E*) and mTMC2 (Fig. 1*F*), as well as the signal of mTMC1 in the membrane fraction assay (*SI Appendix*, Fig. S3*B*). This is in contrast to their apparent absence in the plasmalemma in confocal microscopy analysis (Fig. 1 *B* and *C*). We attribute this discrepancy to the limitations of the techniques employed in our study, specifically nonspecific binding of hydrophobic TMC1/2 membrane proteins to agarose beads in the biotinylation assay (Fig. 1 *E* and *F*) and contamination of intracellular membranous organelles in the membrane fraction assay (*SI Appendix*, Fig. S3*B*). Nevertheless, these biotinylation and membrane fraction assays further verified that the Fyn tag drives the cell-surface expression of TMC1/2 by overcoming their ER retention.

### Ectopic Fyn-mTMC1/2 Alone Generate MS Currents.

**Fig. 2.**
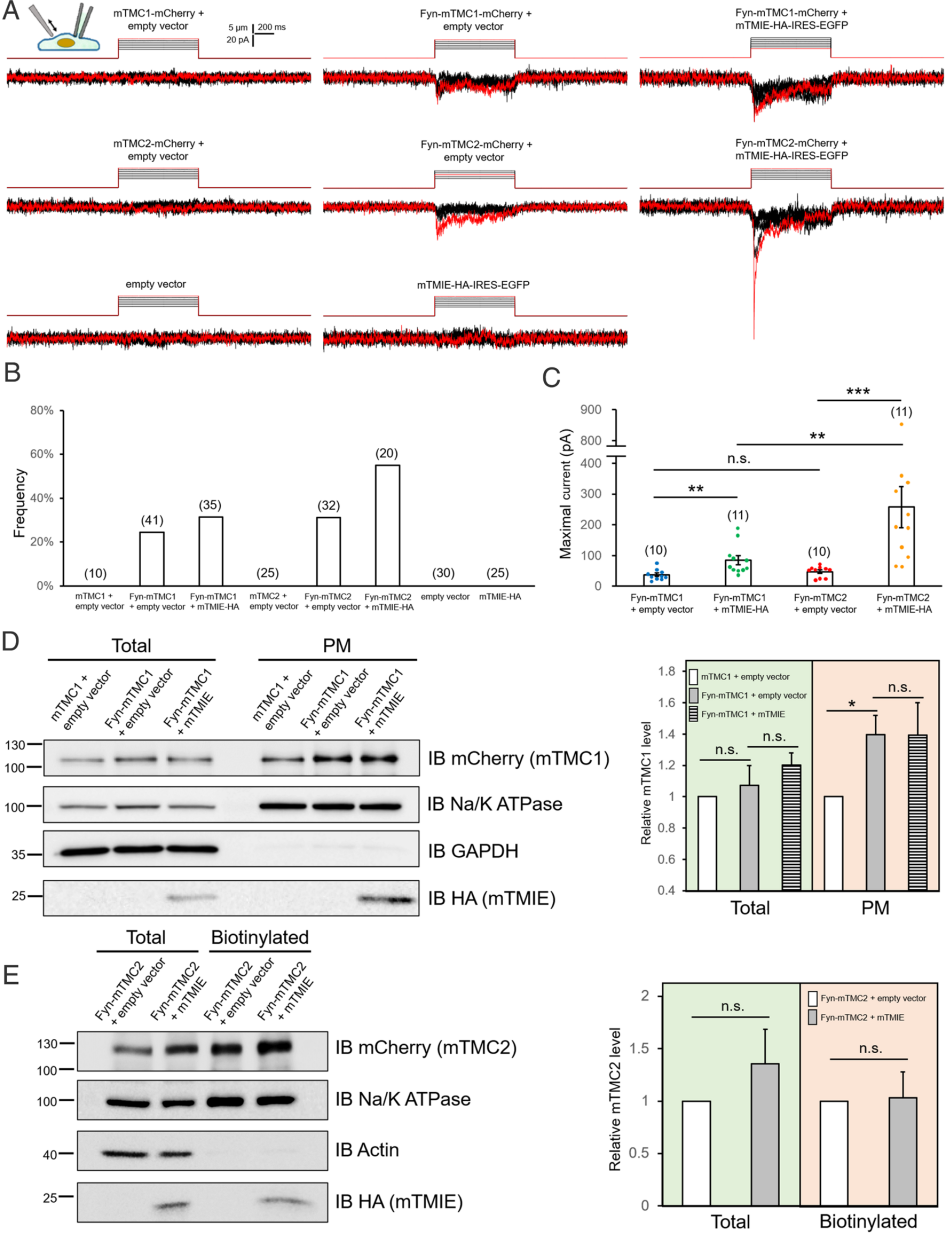
Fyn-TMC1 and Fyn-TMC2 alone generate MS whole-cell currents that are robustly enhanced by TMIE. All experiments were performed in PK-HEK293T cells. (*A*) Representative traces of MS whole-cell currents in cells expressing Fyn-mTMC1/2-mCherry with or without mTMIE-HA. mTMIE-HA was constructed in pcDNA-IRES-EGFP vector. Cells were mechanically stimulated through stepwise indentation (1 μm/step, shown above current traces) at 45° angle. mTMC1-mCherry, mTMC2-mCherry, empty vector, or mTMIE-HA alone failed to generate MS currents. Fyn-TMC1-mCherry and Fyn-TMC2-mCherry generated moderate MS whole-cell currents. Addition of mTMIE robustly increased Fyn-mTMC1/2-mCherry-mediated MS currents. Vm = –80 mV in all panels, except for –90 mV in the panel showing mTMIE-HA alone. Red current traces correspond to the red indentations above. (*B*) Frequency of tested cells that displayed MS whole-cell currents; cells were visually identified based on GFP (for TMIE) and/or mCherry (for TMC1/2) expression. Only cells expressing Fyn-mTMC1/2-mCherry generated MS currents. Number in parenthesis: number of cells tested. (*C*) Summary data of maximal MS current in cells expressing Fyn-mTMC1/2-mCherry with or without mTMIE. Number in parenthesis: total number of cells displaying MS currents. **P ≤ 0.0032; ***P = 0.000040; n.s., not significant. The Mann–Whitney *U* test was used for statistical analysis. (*D*) Total and plasma membrane (PM) fraction of Fyn-TMC1-mCherry assessed using PM isolation assays. *Left* panel: Representative western blots of total and PM fraction of mTMC1-mCherry with or without Fyn tag. Na/K ATPase: loading control for membrane proteins; GAPDH: control for soluble proteins. *Right* panel: Summary data of three experiments similar to that in the *Left* panel. *P = 0.032. Relative intensity was normalized to mTMC1-mCherry without Fyn tag. (*E*) Total (total) and cell-surface (biotinylated) Fyn-mTMC2-mCherry with or without mTMIE. *Left* panel: Representative western blots; *Right* panel: summary data of four experiments similar to that in the *Left* panel. n.s., not significant. Na/K ATPase: loading control. Actin: marker indicating lack of soluble protein contamination in biotinylated fraction.

We used the whole-cell patch-clamp assay to investigate the channel function of Fyn-TMC1/2 in PK-HEK293T cells; these cells are commonly used for studying ectopic MS channels because of their lack of PIEZO1 expression and their minimal basal MS current (16). Fyn-TMC1/2 constructs were generated with a C-terminal fusion of mCherry, which facilitates visual identification of TMC1/2-expressing cells and exerts little effect on TMC1/2 functions (17). Notably, in response to poking (indentation), moderate MS currents were detected in cells that expressed Fyn-mTMC1-mCherry or Fyn-mTMC2-mCherry alone, whereas no MS current was recorded in cells expressing mTMC1/2 or the empty vector (Fig. 2 *A*–*C*). These results suggested that (Fyn-tagged) mTMC1/2 expressed alone, independent of TMIE or other known MT-complex membrane proteins, were sufficient to form functional MS channels. Although these data per se do not exclude the possibility that Fyn-mTMC1/2 serve as regulators and sensitize another MS channel, the MT-channel-like biophysical and pharmacological properties of these MS channels and the effect of putative pore mutation D572N of mTMC2 on its whole-cell and single-channel currents refute this possibility (see below).

### TMIE Robustly Increases Fyn-TMC1/2-Mediated MS Currents.

The Fyn-mTMC1/2-mediated whole-cell MS currents (~37 and ~47 pA for TMC1 and TMC2, respectively) that we recorded in PK-HEK293T cells (Fig. 2) are moderate compared to the MT current of utricular (~200 pA) and cochlear (~400 to 1,700 pA) hair cells (10, 18, 19). We hypothesized that the lack of additional MT-complex components in PK-HEK293T cells at least partly accounts for the modest Fyn-TMC1/2 MS currents. To test this hypothesis, we investigated how Fyn-mTMC1/2 channel function is affected by TMIE because 1) TMIE intimately interacts with the pore-forming transmembrane (TM) helices 6 and 8 of TMC1/2 in *Caenorhabditis elegans* (4, 5) and modulates MT single-channel conductance in the mouse (19); and 2) ectopic TMIE is efficiently expressed at the cell surface in heterologous cells (*SI Appendix*, Fig. S4) (8), which is favorable for examining its effect on TMC1/2 channel activity.

We generated the mTMIE construct in pcDNA-IRES-EGFP vector to allow GFP-based visual identification of mTMIE-expressing cells, and we found that mTMIE robustly increased the Fyn-mTMC1- and Fyn-mTMC2-mediated MS current, by 2.3- and 5.5-fold, respectively (Fig. 2 *A*–*C*). Intriguingly, the mTMIE stimulatory effect on Fyn-mTMC2 was considerably stronger than that on Fyn-mTMC1 (Fig. 2 *A* and *C*), and this also resulted in a markedly higher likelihood of observing Fyn-mTMC2-mediated MS currents than Fyn-mTMC1-mediated MS currents (Fig. 2*B*). Conversely, mTMIE exerted little effect on total and cell-surface levels of Fyn-TMC1/2 in plasma membrane isolation and cell-surface biotinylation assays (Fig. 2 *D* and *E*). These results indicate that mTMIE increased Fyn-mTMC1/2 activity by modulating their channel gating rather than elevating their cell-surface expression.

To further understand the biophysical properties of Fyn-mTMC1/2+mTMIE channels and their differences, we analyzed their activation and inactivation rates: Fyn-mTMC1+mTMIE and Fyn-mTMC2+mTMIE channels showed highly similar activation (τ = 5.4 and 4.8 ms, respectively) inactivation kinetics (τ = 40.0 and 37.3 ms, respectively), which were both moderately slower than those of PIEZO1 (τ = 1.7 ms for activation and 25.5 ms for inactivation), a well-studied bona fide MS ion channel (*SI Appendix*, Fig. S5) (20). The latency in the response of all three channels is attributed to the inherent viscoelastic property of the cell and nonuniform distribution of the cell-surface channels, as observed for other channels (21–23).

### Swapping N Termini of Fyn-mTMC1 and Fyn-mTMC2 Does Not Markedly Affect Their MS Channel Activity.

Upon being Fyn-tagged and fatty acylated, the N-terminal end of mTMC1/2 presumably becomes membrane-associated and sterically constrained (Fig. 1*A*). We sought to determine whether the shorter mTMC1 N terminus (residues 1 to 176) poses greater steric hindrance than the mTMC2 N terminus (1 to 228) and thereby leads to the generation of the smaller Fyn-mTMC1 MS current (Fig. 2). Therefore, we swapped the N termini of Fyn-mTMC1 and Fyn-mTMC2 (*SI Appendix*, Fig. S6*A*) and then measured channel activity, which revealed that the swapping exerted little effect on the activity of Fyn-mTMC1+mTMIE and Fyn-mTMC2+mTMIE (*SI Appendix*, Fig. S6 *B* and *D*) and the frequency of detecting their MS current in cells (*SI Appendix*, Fig. S6*C*). These results indicate that neither the N terminus length and its related steric constraint nor the N terminus composition of mTMC1/2 contributes to the difference in amplitude of their MS channel currents, and TMC1/2 N termini are functionally interchangeable under our experimental conditions.

### Fyn-mTMC2+TMIE Is A Cation-Selective Channel with TMC2 as the Pore-Forming Component in Whole-Cell Studies.

The MS current of mTMC2 was substantially larger than that of mTMC1 (Fig. 2), which makes mTMC2 considerably easier to examine experimentally, and TMC2 is, at least in *C. elegans*, highly similar structurally to TMC1 (4, 5). Thus, we now focused our detailed characterization on Fyn-mTMC2 and Fyn-mTMC2+mTMIE channels.

**Fig. 3.**
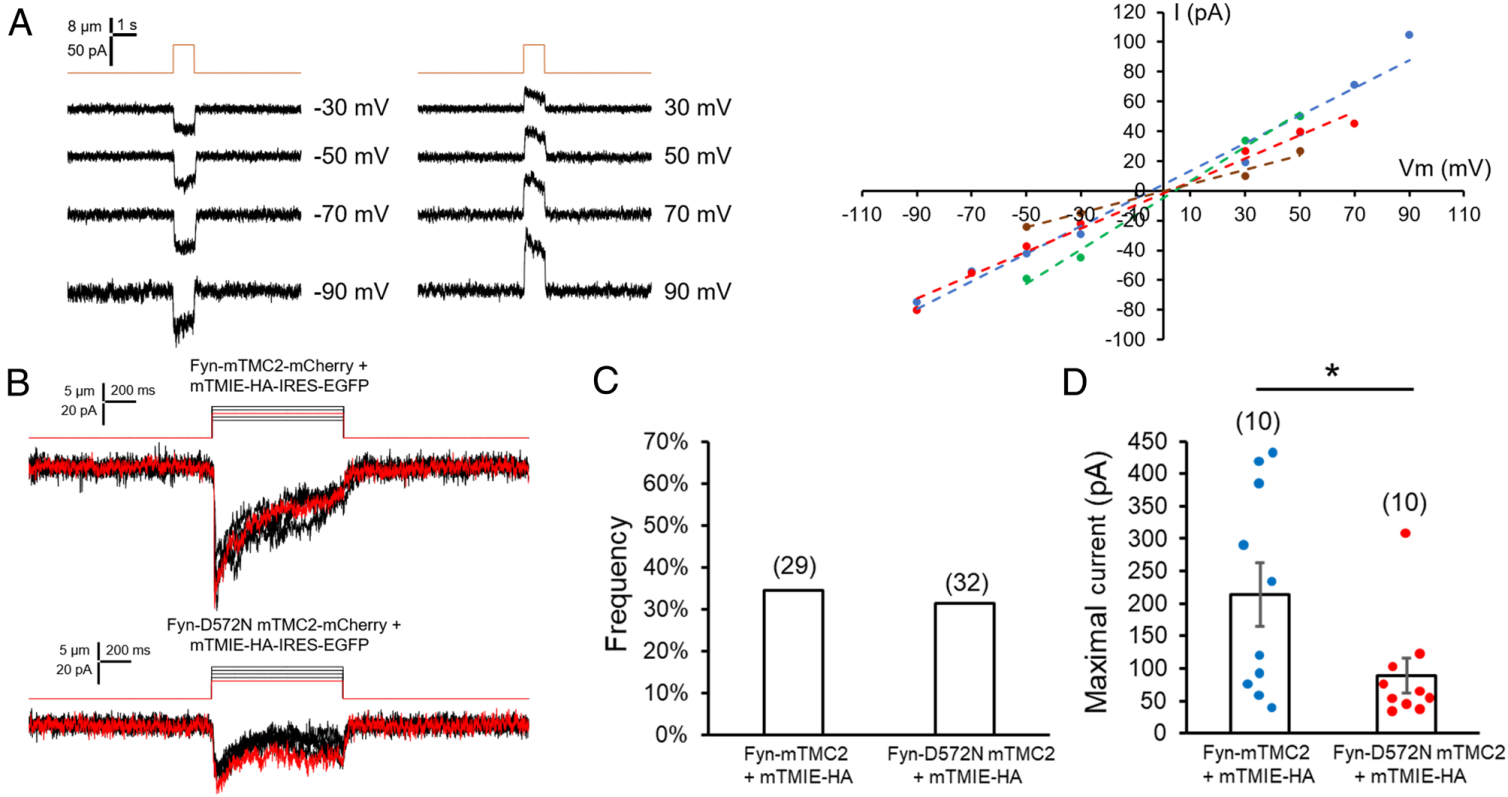
TMC2 is the pore-forming component of the Fyn-mTMC2-mCherry+mTMIE channel. I/V relationship of whole-cell MS currents mediated by Fyn-mTMC2-mCherry+mTMIE. (*A*) *Left*: Representative traces of whole-cell MS current in PK-HEK293T cells coexpressing Fyn-mTMC2-mCherry and mTMIE; *Right*: I/V relationship from four experiments similar to that in the *Left* panel. Ion concentrations in bath and pipette solutions are symmetrical: the bath solution contained (in mM) 137 NaCl, 5.8 KCl, 0.7 NaH_2_PO_4_, 10 HEPES, 1.3 CaCl_2_, 0.9 MgCl_2_, and 5.6 glucose (pH 7.3 with NaOH); the pipette solution contained (in mM) 137 CsCl, 0.1 EGTA, 10 HEPES, and 3.5 MgCl_2_ (pH 7.3 with CsOH). (*B*) Representative traces of MS whole-cell currents in PK-HEK293T cells coexpressing wild-type (*Upper*) or D572N (*Lower*) Fyn-mTMC2-mCherry and mTMIE. Cells were mechanically stimulated through stepwise indentation (1 μm/step, shown above current traces). Vm = −80 mV. Red current traces correspond to the red indentations above. (*C*) Frequency of tested cells that displayed MS whole-cell current. Number in parenthesis: number of cells tested. (*D*) Summary data of maximal MS current in cells expressing wild-type or D572N Fyn-mTMC2-mCherry and mTMIE. Number in parenthesis: total number of cells displaying MS current. *P = 0.035. The Mann–Whitney *U* test was used for statistical analysis.

Under recording conditions similar to those used for studying the MT channel in hair cells (3), the Fyn-mTMC2+mTMIE-mediated MS current displayed a linear I/V relationship (Fig. 3*A*). Moreover, the results of calcium imaging assays showed that mechanical stimulation of Fyn-mTMC2 induced a rise in intracellular Ca^2+^ (*SI Appendix*, Fig. S7), which indicates cation selectivity of the channel. In these assays, we were unable to assess the regulatory effect of TMIE because TMIE expressed alone induced a high basal level of intracellular Ca^2+^, presumably by modulating unknown endogenous, non-MS cation channels as reported previously (24). These biophysical properties of Fyn-mTMC2+mTMIE and Fyn-mTMC2 channels recapitulated those of the MT channel.

To further verify that TMC2 constitutes the conduction pathway of the Fyn-mTMC2+mTMIE MS channel, we generated the D572N mutation in the putative pore-forming region of mTMC2; this mutation is homologous to the D528N mutation in mTMC1, which attenuated the whole-cell current, single-channel conductance, and calcium permeability of the MT channel (19, 25). Notably, the D572N mutation halved the maximal current of the Fyn-mTMC2+mTMIE channel (Fig. 3 *B* and *D*), although its occurrence frequency was similar to that of the wild-type (WT) channel (Fig. 3*C*), and the D572N mutation also reduced the Fyn-mTMC2-mediated intracellular Ca^2+^ increase by 50% (*SI Appendix*, Fig. S7). These results bolster the conclusion that mTMC2 is the pore-forming component of the MS ion channel detected in our study.

### Signal Peptide (SP) Cleavage of mTMIE and Its Effect on Cell-Surface Expression In Vitro.

The TMIE N terminus is predicted to possess a cleavable SP that includes the first TM helix in the hydropathy-plot-based two-pass TM topology of TMIE (*SI Appendix*, Fig. S8*A*), and biochemical and cryo-EM evidence supports the presence of a cleavable SP in hTMIE (24) and CeTMIE (4, 5). Functionally, the first putative TM helix in the SP of zebrafish TMIE appears to be dispensable (26), but the mTMIE SP (the first 27 residues) is suggested to function in MT-channel force sensing or gating (10). To elucidate the role of the SP, we functionally analyzed single-pass TM mTMIE.

First, we experimentally tested whether mTMIE possesses a cleavable SP. By using the HA-mTMIE-Myc construct and 12% SDS gels for clear separation of low-MW proteins in western blots, we found that the mTMIE N terminus is cleaved (*SI Appendix*, Fig. S8*B*), which suggested that mTMIE harbors a cleavable SP, much like hTMIE and CeTMIE. Here, we also noted that the deafness mutation E32G (27), located near the predicted SP cleavage site T28, exerted little effect on SP cleavage (*SI Appendix*, Fig. S8*B*). Second, we generated these two mTMIE mutants: Δ27 mTMIE, to remove the SP for generating a homogenous single-pass TM mTMIE; and T28K mTMIE, to mutate the Thr to the charged residue Lys, which is less preferred by SPase (28), for producing a pure two-pass TM mTMIE (*SI Appendix*, Fig. S8*C*). Importantly, Δ27 mTMIE was expressed predominantly at the cell surface (*SI Appendix*, Fig. S9*A*), whereas T28K mTMIE was trapped in the ER to a greater extent (*SI Appendix*, Fig. S9*A*). Similar to WT mTMIE, the majority of E32G mTMIE localized at the cell surface (*SI Appendix*, Fig. S9*A*), which is consistent with our result that the E32G mutation does not affect TMIE SP cleavage or trafficking (*SI Appendix*, Fig. S8*B*). These results suggest that the TMIE SP affects the cell-surface targeting of the protein. Moreover, deletion of the second TM helix caused cytoplasmic expression of mTMIE (*SI Appendix*, Fig. S9*A*), which supports the notion that the second TM helix is the only TM helix in mature mTMIE.

Agreeing with these results of fluorescence imaging, cell-surface biotinylation assays confirmed that the E32G and Δ27 mutations did not alter the relative cell-surface expression of mTMIE, although the Δ27 mutation decreased the total expression of mTMIE; by contrast, the ΔTM2 and T28K mutations markedly reduced the trafficking of TMIE to the cell surface (*SI Appendix*, Fig. S9*B*).

### Δ27 mTMIE Is as Effective as WT mTMIE in Modulating mTMC2 Gating.

To assess the functional impact of mTMIE SP cleavage, we measured the stimulatory effect of Δ27 and T28K mTMIE on Fyn-mTMC2 (*SI Appendix*, Fig. S9*C*). Notably, Δ27 mTMIE functioned as effectively as WT mTMIE (*SI Appendix*, Fig. S9 *C* and *E*), which generated 50 to 70% single-pass TM mTMIE and certain amount of two-pass TM mTMIE (*SI Appendix*, Fig. S8*A*). These results suggest that single-pass TM mTMIE is sufficient to fully support stimulation of mTMC2 gating. By contrast, T28K mTMIE showed considerably reduced activity (*SI Appendix*, Fig. S9 *C* and *E*), which was in accord with the impaired cell-surface localization of the mutant protein (*SI Appendix*, Fig. S9*B*). These results suggest that in terms of modulating mTMC2 gating, two-pass TM T28K mTMIE, once it reaches the cell surface, functions as effectively as single-pass TM Δ27 mTMIE. Finally, E32G mTMIE stimulated mTMC2 to a comparable level as WT mTMIE (*SI Appendix*, Fig. S9 *C* and *E*), suggesting that the E32G mutation affects neither mTMIE trafficking nor mTMIE modulatory effect on mTMC2, and soluble ΔTM2 mTMIE, as expected, did not affect Fyn-mTMC2 activity (*SI Appendix*, Fig. S9 *C* and *E*). Our results indicate that the N-terminal 27 residues of mTMIE are dispensable for cell-surface expression and modulation of TMC2 gating. However, these results do not exclude other potential functions of the mTMIE N-terminal 27 residues in the MT complex in hair cells, such as in transmitting force from the tip link as proposed previously (10); testing the function of Δ27 mTMIE in hair cells will clarify this matter.

### Palmitoylation of mTMIE Is Essential for Its Modulation of mTMC2 Gating.

**Fig. 4.**
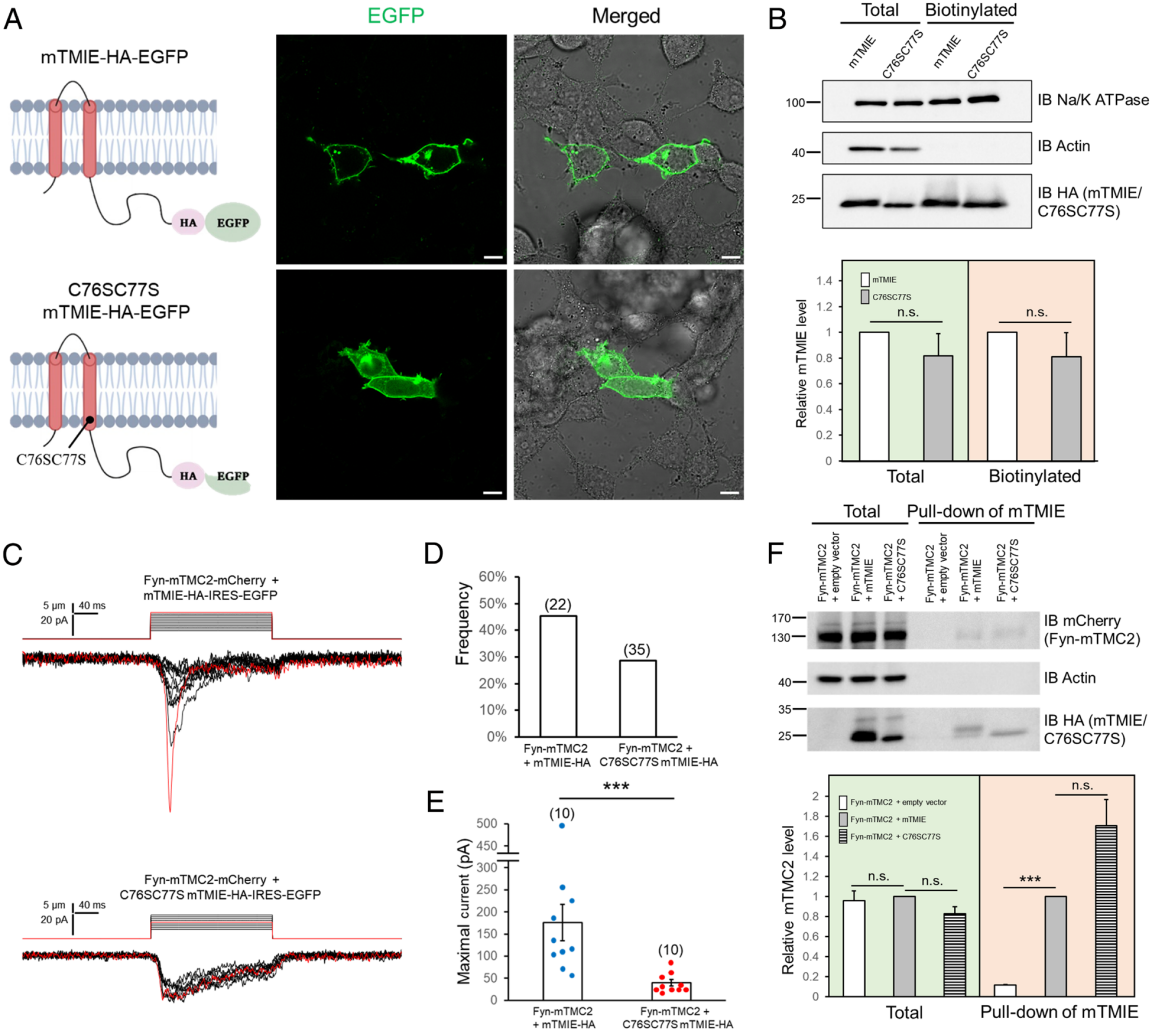
Palmitoylation of TMIE is essential for its regulatory effect on TMC2. WT and C76SC77S mTMIE tagged with HA-EGFP or HA were expressed in PK-HEK293T cells. (*A*) Schematics (*Left*) and fluorescence images (*Right*) of WT (*Upper*) and C76SC77S (*Lower*) mTMIE-HA-EGFP in PK-HEK293T cells. Abundant C76SC77S mTMIE reached the plasma membrane but with slightly more intracellular retention relative to WT mTMIE. (*B*) Total (total) and cell-surface (biotinylated) levels of WT and C76SC77S mTMIE-HA. *Upper* panel: Representative western blot; *Lower* panel, summary data of three experiments similar to that in the *Upper* panel. n.s., not significant. Na/K ATPase: loading control. Actin: marker indicating lack of soluble protein contamination in biotinylated fraction. (*C*) Representative traces of MS whole-cell currents in PK-HEK293T cells expressing Fyn-mTMC2-mCherry with WT or C76SC77S mTMIE-HA. Cells were mechanically stimulated through stepwise indentation (1 μm/step, shown above current traces). Vm = −80 mV. Red current traces correspond to the red indentations above. (*D*) Frequency of tested cells that displayed MS whole-cell currents. Number in parenthesis: number of cells tested. (*E*) Summary data of maximal MS currents in cells expressing Fyn-mTMC2-mCherry with WT or C76SC77S mTMIE-HA. Number in parenthesis: total number of cells displaying MS current. ***P = 0.00044. The Mann–Whitney *U* test was used for statistical analysis. (*F*) Pull-down assay of TMIE and TMC2. PK-HEK293T cells expressing Fyn-mTMC2-mCherry with WT or mutant mTMIE-HA-Avi were subject to pull-down assays with NeutrAvidin beads. *Upper* panel: Representative western blots; *Lower* panel: summary data of three pull-down experiments similar to that in the *Upper*. ***P = 1.33E-9; n.s., not significant.

Recent cryo-EM studies suggest that C43C44 in CeTMIE are palmitoylated and that the palmitoyl groups are inserted near the putative pore region of CeTMC1/2 and interact with hydrophobic residues in the pore-forming TM6 and TM8 (5). The palmitoylations are speculated to mediate the interaction between CeTMC1/2 and CeTMIE and modulate CeTMC1/2 gating, but their function has not been tested experimentally (4, 5). To uncover the role of the palmitoylations, we mutated the two equivalent Cys residues in mTMIE (C76C77) to Ser (Fig. 4*A*); both residues are evolutionarily conserved and predicted to be palmitoylated.

Fluorescence microscopy analysis revealed that C76SC77S mTMIE-GFP was as abundantly expressed at the plasma membrane as WT mTMIE (Fig. 4*A*), and this was further supported by the results of the cell-surface biotinylation assay (Fig. 4*B*). Moreover, in functional analysis, the Fyn-mTMC2+C76SC77S mTMIE channel was observed less frequently than the Fyn-mTMC2+mTMIE channel (Fig. 4*D*), and, importantly, the two Cys-to-Ser mutations nearly abolished the stimulatory effect of mTMIE on Fyn-mTMC2 gating; the amplitude of the MS currents of Fyn-mTMC2+C76SC77S mTMIE (~40 pA, Fig. 4 *C* and *E*) was similar to that of Fyn-mTMC2 alone (~47 pA, Fig. 2). These results indicate that palmitoylation of C76C77 of mTMIE is critical for its regulatory function in mTMC2 gating. Conversely, the C76SC77S mutations did not alter the mTMIE–mTMC2 interaction in pull-down assays (Fig. 4*F*), suggesting that C76C77 palmitoylation affects only the local interaction of mTMIE–mTMC2 that is critical for gating and does not influence the global interaction of the two proteins. The drastic functional reduction resulting from a local, subtle structural change of mTMIE highlights the importance of C76C77 palmitoylation in modulating mTMC2 gating and the specificity of mTMIE in mTMC2 regulation.

### Characterization of Single-Channel Properties and Conductance of Fyn-mTMC1/2+mTMIE Channels.

Once we confirmed that Fyn-mTMC1/2+mTMIE channels replicated the biophysical properties of the MT channel in whole-cell studies, we investigated their single-channel characteristics. This was done using excised, inside–out membrane patches with negative pressure applied via a high-speed pressure clamp.

**Fig. 5.**
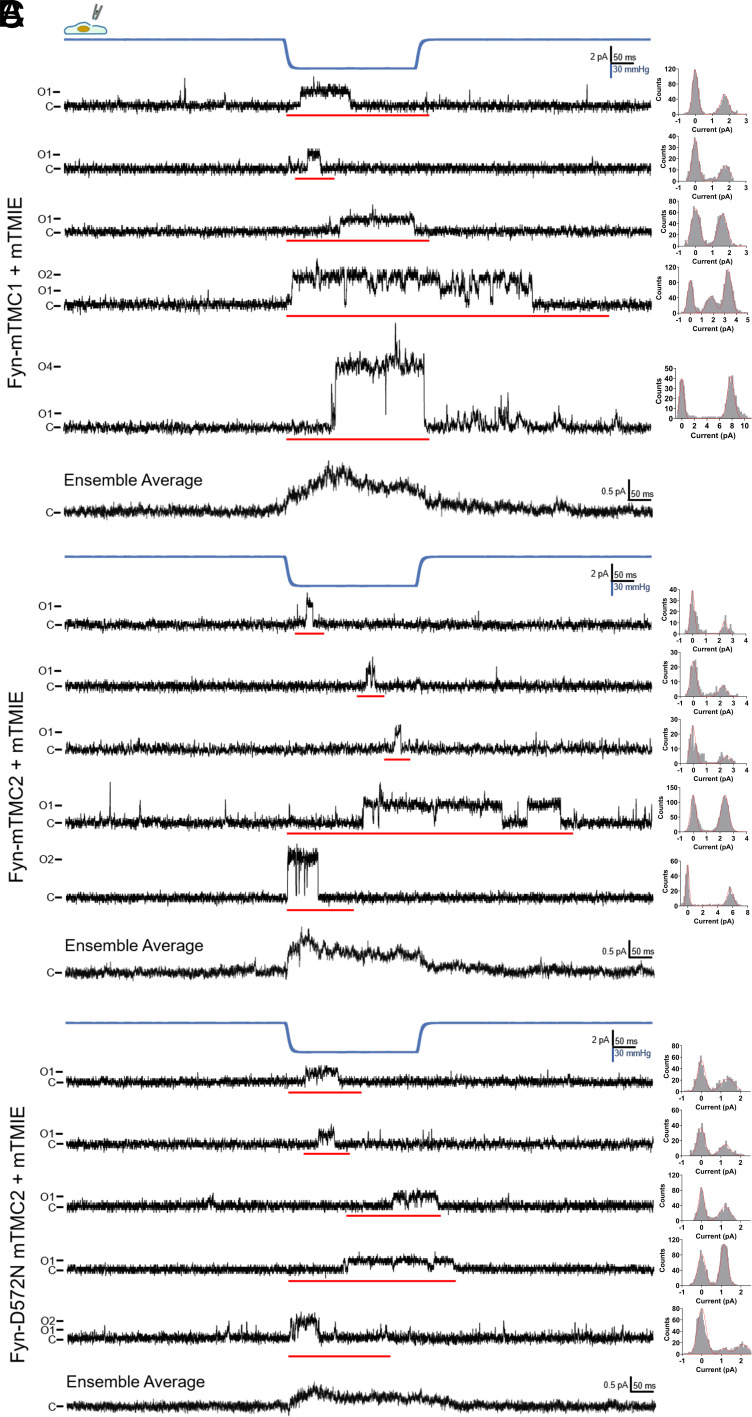
Single-channel recording of Fyn-mTMC1/2-mCherry in excised, inside–out membrane patches. Representative MS single-channel traces from excised, inside–out membrane patches of PK-HEK293T cells coexpressing Fyn-mTMC1-mCherry (*A*), Fyn-mTMC2-mCherry (*B*), and Fyn-D572N-mTMC2-mCherry (*C*) with mTMIE. Bath and pipette solutions contained (in mM) 150 NaCl and 10 HEPES (pH 7.3 with NaOH). Vm = +100 mV. C: closed state of the channel; O1, O2, and O4: open states of one, two, and four channels, respectively. The blue line indicates negative pressure of 60 mmHg applied to membrane patches. All-points amplitude histograms of red-underlined segments of current traces are shown on the *Right*. Ensemble averages represent multiple current traces of Fyn-mTMC1-mCherry+mTMIE (24 traces from 23 cells), Fyn-mTMC2-mCherry+mTMIE (31 traces from 29 cells), and Fyn-D572N-mTMC2-mCherry+mTMIE (22 traces from 22 cells). A low-pass filter at 1 kHz was applied in all experiments presented in Fig. 5.

In the first set of experiments, we applied 1-s mechanical stimulation (negative pressure) and low-pass filtered the data at 0.5 kHz (*SI Appendix*, Fig. S10). In the second set of experiments, we utilized 300-ms mechanical stimulation and low-pass filtering at 1 kHz to more effectively capture and analyze the brief opening events of mTMC1/2 single channels (Fig. 5 and *SI Appendix*, Fig. S11). These short-lived opening characteristics and rapid kinetics have been previously documented for mTMC1/2 channels in hair cells, with a mean open time of approximately 2 ms (10), as well as for other MS channels like Piezo1/2, which exhibit a mean open time of around 13 ms (29, 30).

**Fig. 6.**
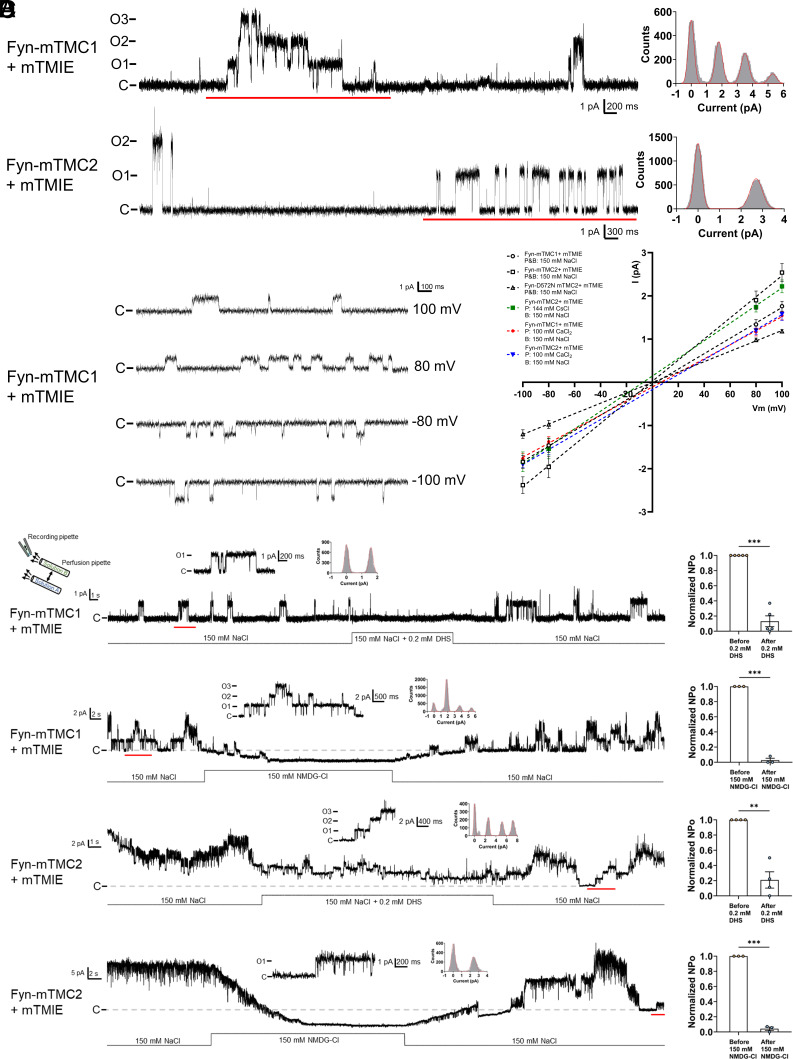
Single channels of Fyn-mTMC1/2-mCherry are sensitive to DHS and cation selective. (*A*) Representative basal activity of MS single-channel recordings from excised, inside–out membrane patches of PK-HEK293T cells coexpressing Fyn-mTMC1-mCherry (18 pS) or Fyn-mTMC2-mCherry (24 pS) with mTMIE. The bath and pipette solutions contained 150 mM NaCl and 10 mM HEPES (pH 7.3 with NaOH). Vm = +100 mV. C: closed state of the channel as shown in this and other panels. *Right*: Amplitude histograms of red-underlined segments of current traces. Red curve: Gaussian fitting. (*B*) Representative single-channel trace of Fyn-mTMC1-mCherry+mTMIE in relative Na/Ca permeability assays. The bath solution in Panels *B* and *C* contained (in mM) 150 NaCl and 10 HEPES (pH 7.3 with NaOH), while the pipette solution contained (in mM) 100 CaCl_2_ and 10 HEPES [pH 7.3 with Ca(OH)_2_]. Summary data from eight similar experiments are present in Panel *C*. (*C*) I-V relations of Fyn-mTMC1/2-mCherry + mTMIE under various ionic conditions in relative Na/Cs/Ca permeability assays. The pipette solution contained (in mM) either 150 NaCl and 10 HEPES (pH 7.3 adjusted with NaOH), 144 CsCl and 10 HEPES (pH 7.3 adjusted with CsOH), or 100 CaCl_2_ and 10 HEPES [pH 7.3 adjusted with Ca(OH)_2_]. P: pipette; B: bath. n = 5 except for n = 8 for the experiment in Panel *B*. Measured reversal potentials were −7.1 mV (Fyn-mTMC2-mCherry) for Cs in the pipette, and +6.6 mV (Fyn-mTMC1-mCherry) or +9.8 mV (Fyn-mTMC2-mCherry) for Ca in the pipette. These values were used to calculate relative Na/Cs/Ca permeability after adjusting for junction potentials, as detailed in the *Materials and Methods* section. (*D*) Effect of DHS and NMDG on the basal activity of Fyn-mTMC1/2 single channels. Black line: changes in bath solution composition. 0.2 mM DHS was applied for approximately 15 s (TMC1) and 20 s (TMC2), and 150 mM NMDG-Cl (replacing 150 mM NaCl) for approximately 30 s for TMC1/2 via a perfusion system. The dotted gray line represents the basal current of the membrane patch, which was decreased by NMDG (see text for further discussion). Short current traces and their corresponding all-points amplitude histograms on top show an expansion of the segment indicated by the red line in the bottom trace, highlighting expanded single-channel events. *Right*: Summary data of normalized NPo 10 s before and during (after) the addition of DHS and NMDG-Cl (excluding the first 4 s, which accounted for the delay in the action of DHS or NMDG-Cl). n = 3 to 5 independent experiments; **P = 0.0051; ***P < 0.001. A 1 kHz low-pass filter was used in all experiments, except for certain experiments in Panel *C*, which utilized a 0.5 kHz low-pass filter.

In PK-HEK293T cells expressing Fyn-mTMC1+mTMIE, Fyn-mTMC2+mTMIE, and Fyn-D572N-mTMC2+mTMIE, we observed MS channels displaying conductance values that were multiples of 18 pS (for Fyn-mTMC1+mTMIE), 24 pS (for Fyn-mTMC2+mTMIE), and 12 pS (for Fyn-D572N-mTMC2+mTMIE), respectively (*SI Appendix*, Fig. S10 and Fig. 5). The mean unitary conductances were 18 ± 2.1 pS for Fyn-mTMC1+mTMIE, 24 ± 2.2 pS for Fyn-mTMC2+mTMIE, and 12 ± 1.4 pS for Fyn-D572N-mTMC2+mTMIE (n = 22 to 31 membrane patches from 22 to 29 cells, Fig. 5). We designated these common divisor conductance values as the unitary conductances for the three channels, as they represented the predominant conductance levels observed in both mechano-stimulated (*SI Appendix*, Fig. S10 and Fig. 5) and basal activities (Fig. 6) of these channels.

The unitary conductance likely reflects the primary conductance state of monomeric mTMC1/2; however, we cannot completely rule out the possibility that these events may represent substates of monomeric mTMC1/2 (*Discussion*). For simplicity, we will refer to these events as the gating of monomeric TMC1/2 channels. The incidence of these MS channels in the tested cells was approximately 33% (5 out of 15) for Fyn-mTMC1+mTMIE, 28% (5 out of 18) for Fyn-mTMC2+mTMIE, and 50% (5 out of 10) for Fyn-D572N-mTMC2+mTMIE (*SI Appendix*, Fig. S10). In contrast, none of the 23 membrane patches from cells expressing empty vectors exhibited these MS channels (*SI Appendix*, Fig. S10).

Notably, the D572N mutation in mTMC2 reduced the unitary conductance from 24 pS to 12 pS (*SI Appendix*, Fig. S10 and Fig. 5), consistent with the whole-cell recording data (Fig. 3 *B*–*D*). This D572N mutation of mTMC2 is similar to the D528N mutation of mTMC1, which also decreased the single-channel conductance of the MT channel (19, 25). These findings suggest that the observed MS channels are mediated by Fyn-mTMC1/2+mTMIE channels. Furthermore, the mean open times for the channels were 3.12 ms for Fyn-mTMC1+ mTMIE, 2.24 ms for Fyn-mTMC2+mTMIE, and 2.01 ms for Fyn-D572N-mTMC2+mTMIE (*SI Appendix*, Fig. S11). The rapid kinetics of these channels are comparable to those of mTMC1/2 channels in hair cells, which have a mean open time of approximately 2 ms (10).

Interestingly, the unitary conductances of mTMC1 (18 pS) and mTMC2 (24 pS) are comparable to those of MS Piezo1/2 channels (20 to 30 pS) (22, 30, 31) and some Ca channels such as Ca_v_1.2 (32, 33), but are smaller than the reported values for mTMC1 (70 to 85 pS) and mTMC2 (51.6 to 58 pS) in hair cells (10, 19). Occasionally, a 40 pS conductance for mTMC1 has been noted in hair cells (19). We hypothesize that these discrepancies may arise from the extent of cooperative gating in monomeric mTMC1/2 (see *Discussion* for more details).

### Fyn-mTMC1/2+mTMIE Single Channels Are Calcium Selective and DHS Sensitive.

In patch-clamp studies, the formation of a giga seal often creates significant surface tension on the membrane patches, which can activate MS channels without any additional mechanical stimulation (34, 35). Interestingly, we also observed considerable basal activity of Fyn-mTMC1/2+mTMIE in certain patches (Fig. 6). The mean conductance values were 18 ± 1.3 pS for Fyn-mTMC1+mTMIE, 24 ± 2.1 pS for Fyn-mTMC2+mTMIE, and 12 ± 0.7 pS for Fyn-D572N-mTMC2+mTMIE (n = 5 for all three groups) (Fig. 6*A*), which were identical to those of the mechano-activated channels (Fig. 5). Because the suction-induced mechanical responses of Fyn-mTMC1/2+mTMIE were not always consistent, it was challenging to assess the effects of any chemicals on these responses. To address this, we utilized the significant basal activity of Fyn-mTMC1/2+mTMIE in some patches to further characterize mTMC1/2 single channels.

We first examined the I-V relationship of Fyn-mTMC1+mTMIE, Fyn-mTMC2+mTMIE, and Fyn-D572N-mTMC2+mTMIE single channels. All three channels exhibited a linear I-V relationship (Fig. 6*C*). In addition, our data suggest that the relative Na/Ca permeability (P_Na_/P_Ca_) of Fyn-mTMC1+mTMIE was 1:2.09, while the relative Na/Cs/Ca permeability (P_Na_/P_Cs_/P_Ca_) of Fyn-mTMC2+mTMIE was 1:0.83:2.55 (Fig. 6 *B* and *C*). These relative permeabilities indicate a preference for Ca over Na and Cs, similar to the characteristics of TMC1/2 channels in hair cells (1).

We also explored the effects of DHS and NMDG. Remarkably, 0.2 mM DHS significantly and reversibly inhibited both Fyn-mTMC1+mTMIE and Fyn-mTMC2+mTMIE (Fig. 6*D*). NMDG fully blocked both channels, reinforcing the idea that Fyn-mTMC1/2+mTMIE channels are cation-selective (Fig. 6*D*). As anticipated, the unitary current of Fyn-mTMC1/2+mTMIE rapidly decreased and then increased when the intracellular solution was replaced with NMDG and subsequently washed out. Additionally, NMDG frequently lowered the baseline current (Fig. 6*D*), likely by blocking the cation-selective leak current through the gigaseals (36).

## Discussion

### Fyn Tag Drives Cell-Surface Expression of Ectopic TMC1/2.

Cell-surface expression of TMC1/2 in heterologous cells has emerged as a technical bottleneck in the unambiguous identification of TMC1/2 as the MT channel. Although reconstitution in artificial liposomes of truncated CmTMC1 and MuTMC2 has been reported, whether full-length mammalian TMC1/2 can form MS channels in heterologous cells has remained unknown. TMC1 cell-surface delivery has been suggested to require the aid of TMC1 binding partners in either the MT complex or the trafficking pathway, but various combinations of these binding partners have failed to drive TMC1 cell-surface expression in studies conducted by us (6) and others (7, 8). Thus, we also investigated whether adding/removing various signal sequences to/from TMC1 can target ectopic TMC1 to the cell-surface membrane. In striking contrast to the failures in our numerous previous attempts, we found here that adding the Fyn lipidation tag to the N terminus of human and mouse TMC1/2 drove the cell-surface expression of these proteins in diverse cell types, as assessed using fluorescence microscopy and cell-surface biotinylation assays (Fig. 1 and *SI Appendix*, Fig. S1). The Fyn tag serves as a signal for myristoylation and palmitoylation, which facilitate stable anchoring of proteins to the plasma membrane and thereby promote their cell-surface expression (12). Although naturally occurring lipidation promotes the cell-surface expression of integral membrane proteins, including several ion channels (11), whether TMC1/2 channels are lipidated in hair cells is unknown, as is whether the lipidation is required for TMC1/2 cell-surface expression. We also observed that some of the expressed Fyn-TMC1/2 remained in intracellular compartments (Fig. 1*B*), probably due to incomplete lipidation of the Fyn tag or overexpression of Fyn-TMC1/2.

We reason that the Fyn tag is unlikely to significantly influence the mechano-activation of mTMC1/2 in our experiments. This conclusion is based on the assumption that the N termini of mTMC1 (residues 1 to 81) and mTMC2 (residues 1 to 129) are disordered loops, as suggested by the Cryo-EM structure of CmTMC1/2 (4, 5) and predictions from AlphaFold 3. Given the relatively small size of the Fyn tag, we anticipate that it would have minimal impact on the structure of these longer disordered loops and, consequently, on the overall conformation of mTMC1/2. However, it remains uncertain whether disordered regions can adopt specific tertiary structures through interactions with other proteins or cellular components within the cell. Therefore, while the Fyn tag may not directly alter the fundamental structural framework of mTMC1/2, we cannot completely rule out the possibility that it could influence their functional interactions or mechano-sensitivity within a cellular context.

### Channel Function of mTMC1/2 in Whole-Cell Studies.

Although early studies suggested that both mTMC1/2 and mTMIE are pore-forming subunits of the MT channel (1–3, 10), a recent study suggested that the MET current, albeit substantially diminished, can still be recorded in TMIE knockout mice, which argues against an indispensable role of TMIE in the pore formation of the MT channel (19). The results of previous reconstitution experiments have suggested that truncated nonmammalian TMC1/2 alone form MS channels, but whether full-length mammalian TMC1/2 can form channels by themselves has been unclear. Our results from both whole-cell patch-clamp and calcium imaging assays (Fig. 2 and *SI Appendix*, Fig. S7) suggest that full-length mTMC1/2 alone can form channels and generate MS currents and calcium influx. Notably, PK-HEK293T cells lack all the other known MT-complex proteins, although they likely express CIB2, according to an mRNA database search. Soluble CIB2 might not directly contribute to the pore formation of the channel, but it could potentially affect TMC1/2 gating in PK-HEK293T cells; further investigation is necessary to address this question.

Interestingly, we found that mTMC2, in the presence of mTMIE, generated a larger MS current than mTMC1. Although our single channel data showed that Fyn-mTMC2 has a unitary conductance ~30% larger than Fyn-mTMC1 (Fig. 5 and *SI Appendix*, Fig. S10), the difference is insufficient to account for the difference in the MS whole-cell currents. Alternatively, a smaller TMC1 current could result from the lack of certain TMC1-specific regulatory proteins in PK-HEK293T cells. For example, LHFPL5 is required for the native channel function of TMC1 but not TMC2 (37).

### TMIE Function and Palmitoylation.

Previous studies have suggested that mTMIE assists mTMC1 targeting to the transduction site and transmits force from the tip link to mTMC1/2 in hair cells (8, 10), and mTMIE has also been suggested to form a part of the pore of the MET channel (10). However, our results indicate that mTMIE is dispensable for the pore formation of mTMC1/2 because mTMC1/2 alone were able to form MS channels (Fig. 2 and *SI Appendix*, Fig. S7), which agrees with a recent functional study in TMIE knockout hair cells (19). Conversely, we found that mTMIE serves as a critical modulator of TMC1/2 channel gating.

The single-channel conductance of the TMC1-mediated MT channel was previously shown to be reduced by 25% in TMIE knockout hair cells in the mouse (19). However, the stimulatory effect of mTMIE on mTMC1/2 gating we measured in this study was ~2.3 to 5.5-fold (Fig. 2), which is substantially higher than the previously reported 25% change in single-channel conductance (19). Thus, TMIE might contribute to the force sensing of TMC1/2 channels in addition to modulating single-channel conductance, considering that mTMIE exerted no effect on Fyn-mTMC1/2 cell-surface expression (Fig. 2). Notably, we found that the palmitoylation of mTMIE at C76C77 is critical for its regulatory function (Fig. 4): Mutating C76C77 and removing the potential palmitoylation eliminated the mTMIE stimulation of mTMC2. These results indicate a key role of potential C76C77 palmitoylation in the force sensing of TMC1/2.

The amplitude of the MS whole-cell current mediated by Fyn-mTMC1/2+mTMIE was ~100 to 300 pA (Fig. 2), which is considerably smaller than the native MT current recorded in cochlear hair cells (~400 to 1,700 pA) but is comparable to that in utricular cells (~200 pA) (10, 18, 19). Thus, comparing the amplitudes of these currents directly can be challenging and perhaps not highly informative because of the differences in the stimulation modes and cellular contexts between PK-HEK293T and hair cells (see below).

### Single-Channel Conductance and Cooperative Gating of TMC1/2.

We characterized the single-channel properties of Fyn-mTMC1/2+mTMIE in excised, inside–out membrane patches of PK-HEK293T cells. These channels exhibited responsiveness to membrane stretch, fast kinetics, linear I-V relationships, Ca selectivity, DHS sensitivity, and NMDG sensitivity (Figs. 5 and 6 and *SI Appendix*, Figs. S10 and S11). These biophysical and pharmacological properties, similar to the whole-cell currents (Figs. 2 and 3), mirror those of the MT channel. Notably, the putative pore mutation D572N of mTMC2 halved the unitary conductance (*SI Appendix*, Fig. S10 and Fig. 5), reinforcing the idea that TMC2 constitutes the conduction pathway of the Fyn-mTMC2+mTMIE MS channel.

Interestingly, the predominant conductances of Fyn-mTMC1+ mTMIE and Fyn-mTMC2+mTMIE were 18 pS and 24 pS, respectively (Figs. 5 and 6*A*), likely reflecting the gating of monomeric mTMC1/2. These values are lower than the reported unitary conductance of mTMC1 (70 to 85 pS) and mTMC2 (52 to 58 pS) in hair cells (10, 19). We hypothesize that these discrepancies result from the cooperative gating of monomeric mTMC1/2, as evident in both mechano-stimulated and basal activities (Figs. 5 and 6). The 72 pS mTMC1 and 48 pS mTMC2 observed in Figs. 5 and 6 presumably reflect the cooperative gating of four monomeric mTMC1 or two monomeric mTMC2.

Several factors influence cooperative channel gating, including channel oligomerization, clustering, subunit composition and arrangement, lipid environment, cytoskeletal interaction, permeant ions of channels (such as Ca), and posttranslational modifications (38). Although the composition and organization of the MT complex are not fully understood, mTMC1/2 exist in a distinctly different protein and lipid environment than Fyn-TMC1/2. For instance, PCDH15 and LHFPL5 are absent in PK-HEK293T cells; a previous study suggested the clustering of 8 to 20 TMC1 monomers at each transduction site (39); and PIP2, a known regulator of channel cooperative gating (40), is highly enriched at the transduction site (41, 42). Interestingly, previous studies suggested the possible cooperative gating of TMC1 (19, 43). Presumably, the context of hair cells, including the assembly of the MT complex and lipid environment, favor cooperative gating of four monomeric mTMC1 and two monomeric mTMC2, which are manifested as approximately 70 to 80 pS and 52 to 58 pS channels (10, 19).

The strong cooperative gating of mTMC1/2 channels, along with the low signal-to-noise ratio in hair cell recordings from previous studies, might obscure the 18 pS monomeric mTMC1 and 24 pS monomeric mTMC2 (10, 19, 44). Single-channel recordings were conducted in whole-cell mode due to difficulties in analyzing mTMC1/2 channels in cell-attached patches (10, 19, 44). In addition, low-pass filtering at 2 to 5 kHz increased the noise level compared to our study’s 0.5 to 1 kHz cutoff. Previous studies indicated a noise level of about 3 pA (10, 19, 44), significantly higher than our measurements (<1 pA, Figs. 5 and 6). Thus, the 18 pS or 24 pS currents (1.5 or 1.9 pA at −80 mV) would be challenging to resolve against this noise level, despite occasional detection of larger 40 pS mTMC1 channels (19). Further experimental investigation is necessary to determine whether TMC1/2 exhibit cooperative gating in hair cells. Cooperative channel gating plays important physiological roles, including enhancing peak current, activation and inactivation rates, and response frequency. These factors are crucial for the physiological function of TMC1/2 channels in processing rapid sound stimulation.

### Calcium Permeability of Fyn-mTMC1/2 Channels.

We examined the P_Na_/P_Cs_/P_Ca_ of Fyn-mTMC1/2+mTMIE (Fig. 6). Our data suggested a P_Na_/P_Ca_ of 1:2.09 for Fyn-mTMC1+mTMIE, implying a P_Cs_/P_Ca_ of 1:2.54 [assuming a P_Na_/P_Cs_ of 1:0.82 (45)]. This value is close to or smaller than the previously reported values of ~2.7 (46) or 3.7 to 4.4 (1) for mTMC1 in hair cells. The P_Na_/P_Cs_/P_Ca_ of Fyn-mTMC2+mTMIE was 1:0.83:2.55, indicating a P_Cs_/P_Ca_ of 1:3.06. This value is smaller than the reported range of 5 to 6.3 for mTMC2 channel in hair cells (1, 2). One possible reason for the discrepancies is the absence of certain regulatory proteins in PK-HEK293T cells. For instance, the accessory protein STIM1 significantly increased Ca permeability of the CRAC channel (47). In addition, MinK, a single-pass TM protein, altered ion selectivity and open channel block of a slowly activating potassium current in *Xenopus* oocytes (48). Furthermore, the R82C mutation of mTMIE reduced calcium permeability of MET current by 30% (10), suggesting that Ca permeability of mTMC1/2 can be modulated by accessory proteins. It is unclear how other known or unknown accessory proteins such as LHFPL5, PCDH15, and CIB2 modulate Ca permeability of mTMC1/2. In addition, lipids can also alter the ion selectivity of ion channels (49). Presumably, the lipid environment in PK-HEK293T cells and hair cells are very different (41, 42).

### Other MT-Complex Components.

We also attempted to test how TMC1/2 channel activity is affected by LHFPL5, another key MT-complex component. LHFPL5 is suggested to directly to transmit force from the tip link to TMC1/2 and regulate their protein stability and targeting to the transduction site (6, 50, 51) rather than to modulate the single-channel conductance of the MT channel (19). However, LHFPL5 is also trapped in the ER (6, 50), and adding the Fyn tag to LHFPL5 failed to target it to the cell surface. One possibility is that the short N terminus of LHFPL5 presents a steric constraint against effective palmitoylation of the Fyn tag and/or subsequent insertion into the membrane. In preliminary experiments, we also tested how ectopic CIB2 expression affects TMC1/2 channel activity, but we did not observe any effect of CIB2. We speculate that abundant expression of endogenous CIB2 in PK-HEK293T cells might mask the effect of exogenous CIB2. CIB2, unlike other MT-complex components, is expressed in cell types other than hair cells.

### Summary.

We have reported several notable findings: 1) A Fyn lipidation tag enhances cell-surface expression of mouse and human TMC1/2, helping to overcome longstanding technical challenges in characterizing TMC1/2 in heterologous cells; 2) Full-length mTMC1/2, when expressed alone, function as MS channels in whole-cell patch-clamp assays, supporting the idea that TMC1/2 are the sole pore-forming subunits of the MT channel; 3) mTMIE significantly boosts mTMC1/2 channel activity without affecting their cell-surface expression; 4) The N-terminal 27 residues of mTMIE are not necessary for its stimulation of TMC1/2, while palmitoylation at C76C77 is crucial for its regulatory role; and 5) mTMC1/2+mTMIE form 18 pS and 24 pS single channels, respectively, which exhibit biophysical and pharmacological properties similar to those of the MT channel.

These findings provide insights into the function and regulation of TMC1/2 and TMIE. Furthermore, our in vitro functional assay for TMC1/2 and TMIE will greatly facilitate future functional and structural studies of the MT complex and has the potential to be developed into a medium- or high-throughput assay for screening specific high-affinity drugs targeting TMC1/2 and other MT-complex components. This is crucial given the current scarcity of such drugs, which are vital for basic research and clinical applications in the field of hearing research.

## Materials and Methods

For functional studies, the N terminus of mTMC1/2 was fused with the Fyn tag, a membrane-anchoring motif (MGCVQCKDKEA), to enhance cell-surface expression in PIEZO1-knockout HEK293T (PK-HEK293T) cells. In whole-cell patch-clamp assays, mechanical stimulation was applied by poking the cells with a fire-polished glass probe, with displacement controlled by a piezoelectric system. For single-channel studies in excised, inside–out membrane patches, negative pressure (suction) was applied at the back of the pipette using a High-Speed Pressure Clamp. Please refer to *SI Appendix* for complete details.

## Supplementary Material

Appendix 01 (PDF)

## Data Availability

All study data are included in the article and/or *SI Appendix*.
