# Supplementary material for: Ectopic mouse TMC1 and TMC2 alone form mechanosensitive channels that are potently modulated by TMIE
Source: Proc Natl Acad Sci U S A. 2025 Feb 25;122(9):e2403141122. doi: 10.1073/pnas.2403141122 (PMC11892609; doi:10.1073/pnas.2403141122)
Supplement: Supplementary file 1 — Appendix 01 (PDF) [file pnas.2403141122.sapp.pdf]

Supplementary materials and methods

Cell culture and transfection

PK-HEK293T (PIEZO1-knockout HEK293T) cell line was generously provided by Dr. Bailong Xiao (Tsinghua University). HEK293T, 3T3, and COS7 cell lines were purchased from American Type Culture Collection (ATCC); the cell lines were presumably authenticated by ATCC and were not further authenticated in this study. All cell lines were cultured in Dulbecco's Modified Eagle Medium (DMEM) supplemented with 10% fetal bovine serum and 100 mg/L ampicillin in an atmosphere of 95% air/5% CO_2 at 37°C. Cells were transfected using polyethylenimine (23966-2; Polysciences), as previously described (1).

Immunostaining and fluorescence imaging

Cells were digested with trypsin and re-plated on specialized confocal dishes. For live imaging, cells were examined under a confocal microscope (SP8 LIGHTNING, Leica Microsystems) at room temperature after 24 h of transfection with various plasmids and subsequent change of the culture medium to Opti-MEM (31985088, Thermo Fisher Scientific). Cell nuclei were stained by incubating cells with Hoechst 33258 dye for 15 min and then washing twice with Opti-MEM before imaging. For immunostaining, transfected cells were first washed twice with PBS, fixed with 4% (w/v) paraformaldehyde (PFA) at room temperature for 30 min, washed thrice with PBS, and permeabilized and blocked with PBS containing 4% (w/v) BSA and 0.1% (v/v) Triton X-100 for 30 min. Next, the cells were washed thrice with PBS, incubated at room temperature for 2 h with PBS containing 4% (w/v) BSA, 0.1% (v/v) Tween-20, and a primary antibody (diluted 1:1000), washed thrice more with PBS, and incubated at room temperature in the dark for 1 h with PBS containing 4% (w/v) BSA, 0.1% (v/v) Tween-20, and a fluorophore-labeled secondary antibody (1:5000). Lastly, the cells were washed three times with PBS and subsequently prepared for fluorescence confocal imaging.

Confocal images were acquired using a Leica SP8 microscope equipped with a 63× oil objective lens and a numerical aperture of 1.4. The pinhole size was set to 1.00 AU, and the pixel dimensions in the X-Y plane were 2048 × 2048. The calculated widths of the point spread function were 226.57 nm at an emission wavelength of 520 nm and 252.71 nm at 580 nm.

For colocalization analysis, we first adjusted the threshold using the "Auto Threshold" feature in ImageJ software. The region of interest (ROI) was defined as the plasma membrane area, selected using the "MorphoLibJ" plugin. We then employed the "Coloc 2" plugin to conduct the colocalization analysis.

Western blotting

Western blotting was performed as per published procedures (2). Briefly, protein samples were mixed with 20% (v/v) 6×SDS loading buffer, separated by electrophoresis, and transferred to PVDF membranes, which were blocked with 5% (w/v) skimmed milk in TBST buffer for 30 min at room temperature. Primary antibodies (1:1000) were applied overnight at 4°C or for 2 h at room temperature, and after washing, horseradish peroxidase-conjugated secondary antibodies (1:5000) were applied for 2 h at room temperature. Lastly, membranes were washed and immunostaining was visualized using enhanced chemiluminescence substrates.

Cell-surface biotinylation assay

Cell-surface biotinylation was performed using previously described procedures (3, 4) with minor modifications. PK-HEK293T cells were transfected with various plasmids and incubated for 24 h, washed twice with ice-cold PBS-CM (PBS containing 2.5 mM $CaCl_2$ and 1 mM $MgCl_2$), and incubated with 1 mg/mL cell-surface biotinylation reagent in PBS-CM in the dark at 4°C for 45 min; EZ-Link™ Sulfo-NHS-LC-Biotin and EZ-Link™ Sulfo-NHS-SS-Biotin (Cat.# 21335 and 21331, Thermo Fisher Scientific) were used for Fyn-TMC1/2 and WT/mutant TMIE, respectively. Subsequently, the cells were washed once with ice-cold PBS-CM, incubated for 5 min on ice with ice-cold PBS-CM containing 50 mM Tris to neutralize any residual biotinylation agent, washed twice with ice-cold PBS-CM, and lysed with RIPA buffer containing cOmplete Mini protease-inhibitor cocktail (1183615300, Sigma-Aldrich). Next, after removing cell debris by centrifugation at 15,700 × *g* at 4°C for 10 min, the obtained lysate was mixed with pre-blocked NeutrAvidin agarose beads (29201, Thermo fisher Scientific) at 4°C overnight with gentle shaking, and the beads were then washed six times with ice-cold PBS containing 0.2% (v/v) Triton X-100 and eluted in 1× SDS loading buffer for western blotting.

Pull-down assays

To perform the pull-down assay of mTMIE and mTMC2, we added an HA-Avi tag at the mTMIE C-terminus in a bicistronic pcDNA3 vector, which contains an internal ribosomal-entry site for independent expression of codon-optimized hBirA biotin ligase and thereby enables biotinylation of Avi-tagged proteins (1). PK-HEK293T cells were transfected with Fyn-mTMC2 and mTMIE plasmids (w/w ratio: 3:2), and at 24 h after transfection, the cells were washed twice with ice-cold PBS and then lysed using a solution containing 150 mM NaCl, 10 mM HEPES (pH 7.3 with NaOH), 0.5% (v/v) NP-40, and cOmplete Mini protease-inhibitor cocktail. Cells were lysed through brief ultrasonication followed by gentle stirring at 4°C for 1 h, and after centrifuging the lysate at 15,700 × *g* at 4°C for 10 min to remove cellular debris, and the resulting supernatant was incubated with pre-washed NeutrAvidin agarose beads at 4°C overnight. Lastly, the beads were washed thrice with ice-cold PBS containing 0.2% (v/v) NP-40 to remove non-specifically bound proteins and then used for western blotting.

Whole-cell poking and patch-clamp assays

Briefly, PK-HEK293T cells were incubated for 24 h after transfection before performing the whole-cell patch-clamp assay at room temperature. For co-transfection of two plasmids, the w/w ratio of TMC1/2 plasmids and TMIE (or TMIE mutant) plasmid was 3:1. The bath solution contained (in mM) 137 NaCl, 5.8 KCl, 0.7 NaH_2PO_4, 10 HEPES, 1.3 $CaCl_2$, 0.9 $MgCl_2$, and 5.6 glucose (pH 7.3 with NaOH); the pipette solution contained (in mM) 137 CsCl, 0.1 EGTA, 10 HEPES, 3.5 $MgCl_2$ (pH 7.3 with CsOH). The recording glass pipettes (1B150-4 Borosilicate Glass Capillaries, World Precision Instruments) were pulled and polished using a pipette puller (DMZ, Zeitz Instruments) and featured a resistance of 3–5 MΩ. The mechanical stimulus was applied by poking cells with a fire-polished glass probe with a tip diameter of 2–3 μm. The displacement of the glass probe was controlled by a piezoelectric system (P-601 PiezoMove Linear Actuator/E-625 Piezo Servo Controller, Physik Instrumente) that was under the command of a digitizer (Axon™ Digidata® 1550B, Axon Instruments). The glass probe was set against the cell membrane at a 45° angle and each step of the increment was 1 μm. Data were acquired using an Axopatch 200B amplifier and Axon™ Digidata® 1550B with Axon Clampex 10.7 software. The data were sampled at 10 kHz and filtered at 2 kHz. Data were analyzed with Clampfit 10.7 (Molecular

Devices). For the measurement of the inactivation time constant, all current responses, except for traces with a noticeable shift in baseline, were individually calculated and averaged.

Single-channel recording and analysis

For single-channel recording of excised, inside-out membrane patches, both the bath and pipette solutions contained (in mM) 150 NaCl, 10 HEPES (pH 7.3 with NaOH). The resistance of the recording glass pipette was 4–6 MΩ, and the negative pressure (suction) was applied in the back of the pipette by a High Speed Pressure Clamp (HSPC-2-SB, ALA Scientific Instruments). Dihydrostreptomycin (DHS) and NMDG-Cl were applied to and removed from the membrane patches using a fast-step perfusion system (SF-77B, Warner Instrument). The data were sampled at 10 kHz and low-pass filtered at 500 Hz or 1 kHz. Vm was often maintained at positive voltages, where Fyn-mTMC1/2 channels exhibited a higher open probability, similar to Piezo1 channel (5).

The relative permeability of Na, Cs and Ca of Fyn-mTMC2-mCherry+mTMIE channel was determined by performing excised, inside-out single-channel recording and measuring reversal potentials. The bath solution used for these experiments contained (in mM) 150 NaCl and 10 HEPES (pH 7.3 with NaOH). For measuring the relative Cs permeability, the pipette solution contained (in mM) 144 CsCl and 10 HEPES (pH 7.3 with CsOH); for measuring the relative Ca permeability, the pipette solution contained (in mM) 100 $CaCl_2$ and 10 HEPES [pH 7.3 with $Ca(OH)_2$]. The reversal potentials were corrected for -1.3 mV and -4.1 mV junction potentials with Cs and Ca in the pipette, respectively. The permeability ratio, P_{Cs}/P_{Na}, was calculated as described previously (6).

$$P_{Cs}/P_{Na} = [Na^{+}] \cdot exp(V_{REV}F/RT)/[Cs^{+}]$$

The permeability ratio, P_{Ca}/P_{Na}, was also calculated as described previously (7).

$$P_{Ca}/P_{Na} = \{a_1[Na^{+}]/4a_2[Ca^{2+}]\} \cdot \{exp(V_{REV}F/RT)\} \cdot \{1 + exp(V_{REV}F/RT)\}$$

where V_{REV} means reversal potential, RT/F has its usual meaning with a value at room temperature of 25.7 mV, and a_1 and a_2 are the published activity coefficients for Na^{+} (8) and Ca^{2+} (9), respectively.

Calcium imaging assays

Fluo-4-AM (F14201, Thermo Fisher Scientific) was used as the calcium indicator (excitation: 494 nm; emission: 506 nm). After transfection, PK-HEK293T cells were incubated for 24 h, washed once with PBS, and then incubated with 5 μM Fluo-4-AM in Opti-MEM at 37°C in the dark for 30 min. After washing once with PBS and incubating for another 30 min in Opti-MEM, cells were imaged in the bath solution used for whole-cell patch-clamp assays. Indentation was used to mechanically activate Fyn-mTMC2, Fyn-D572N mTMC2, and mPIEZO1 channels in PK-HEK293T cells and induce a rise in intracellular calcium. Calcium signals were captured with an EMCCD camera (Andor, Oxford Instruments) and analyzed using NIS-Elements AR software (Nikon) and ImageJ.

Statistics

Means ± SEM are presented in all figures. Data normality was tested using the D'Agostino-Pearson omnibus normality test or Shapiro-Wilk normality test. For normally distributed data, statistical significance was assessed using unpaired two-tailed Student's *t* test, unless indicated otherwise; for non-normally distributed data, Mann-Whitney U test was applied. Differences were considered significant at $p < 0.05$.

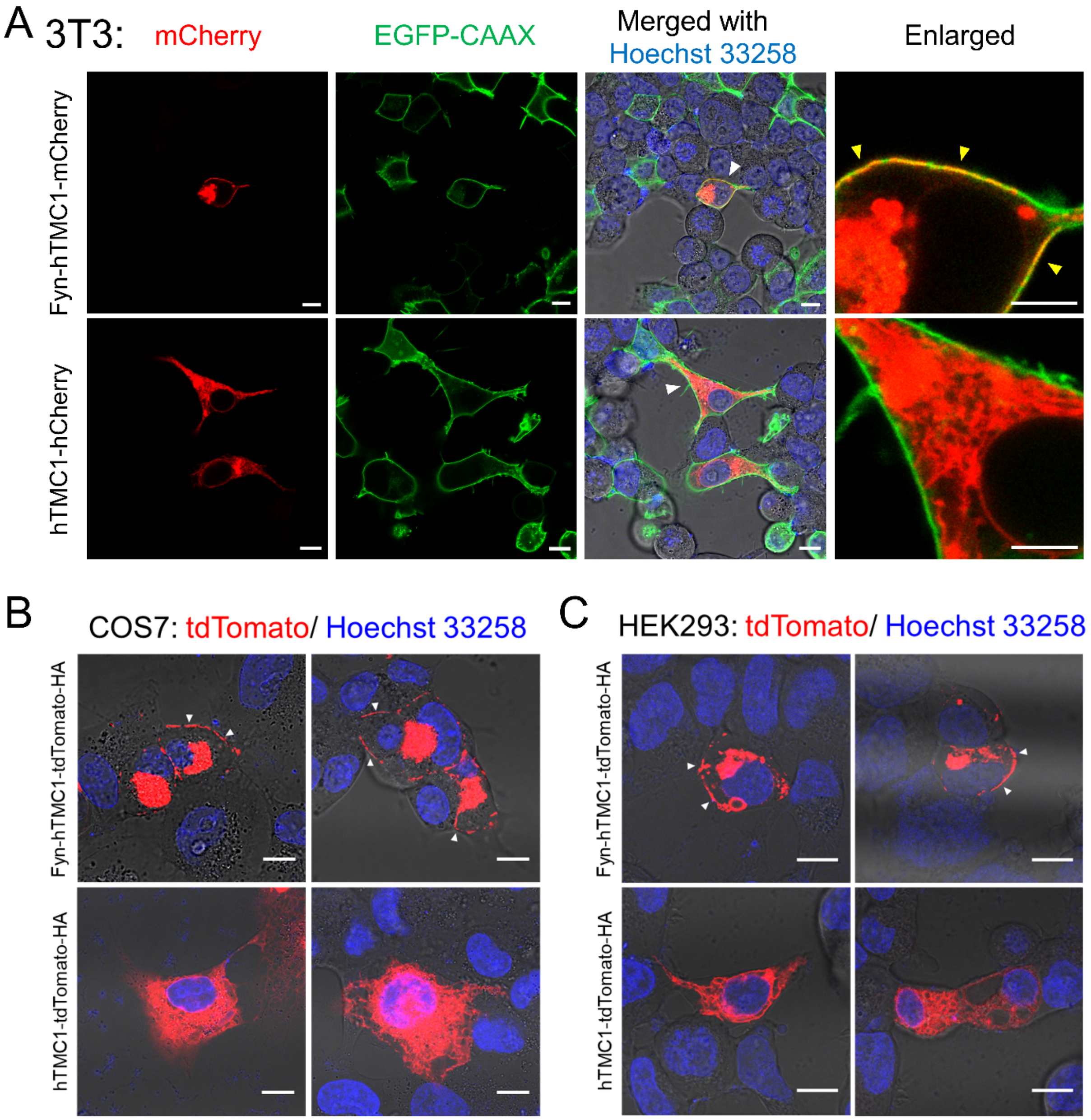

Supplementary Figure 1. Fyn tag drives cell-surface expression of ectopic human TMC1 in several cell types besides PK-HEK293T cells. (**A**) Confocal images of 3T3 cells expressing hTMC1-mCherry (red) with or without N-terminal Fyn tag. EGFP-CAAX (green): cell-surface marker; Hoechst 33258 staining (blue): nuclei (here and in Panels B-C). Last column: enlargement of area indicated by white arrowhead in 3rd column; yellow arrowhead: overlap of Fyn-TMC1-mCherry with EGFP-CAAX. Scale bars: 10 μm, except for 5 μm in last column. (**B-C**) Confocal images of COS7 (**B**) and HEK293 (**C**) cells expressing hTMC1-tdTomato-HA (red) with or without Fyn tag. White arrowhead: cell-surface expression of Fyn-hTMC1-tdTomato-HA. Scale bars: 10 μm.

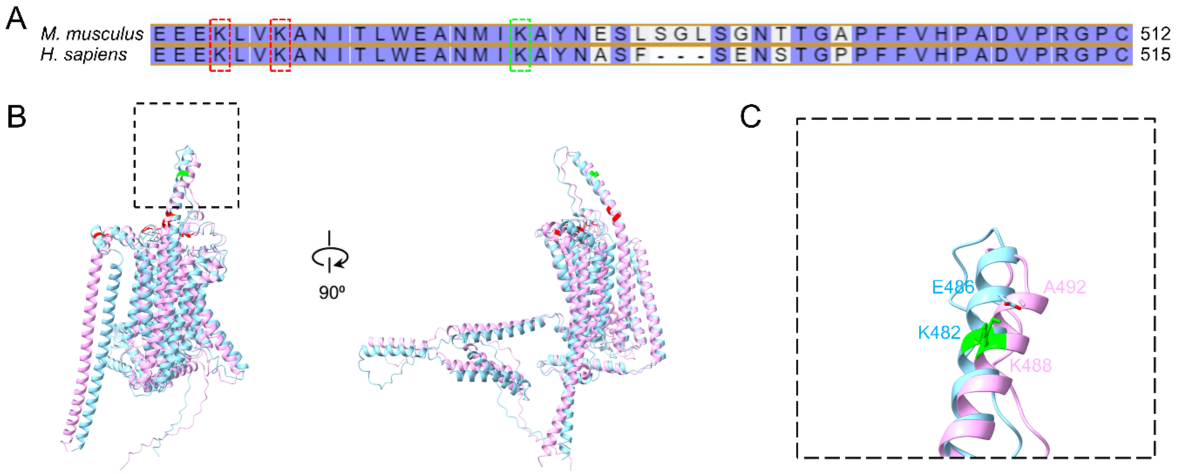

Supplementary Figure 2. Modeling of accessibility of extracellular lysines in mTMC1 and hTMC1. (A) Primary sequences of 3rd extracellular loop of mTMC1 and hTMC1. Identical residues are blue-highlighted. Accessible K482 in mTMC1 and K488 in hTMC1 are highlighted by green dotted boxes, and other two lysines red-dotted boxes. **(B)** AlphaFold 2 modeling of mTMC1(cyan) and hTMC1 (violet) 3-D structures. Green highlighted: K482 in mTMC1 and K488 in hTMC1; red-lighted: all other extracellular lysine residues; dotted box: 3rd extracellular loop. **(C)** Detailed view of the dotted box of Panel B. In mTMC1, E486 forms a salt bridge with K482, while in hTMC1, A492 is equivalent to E486.

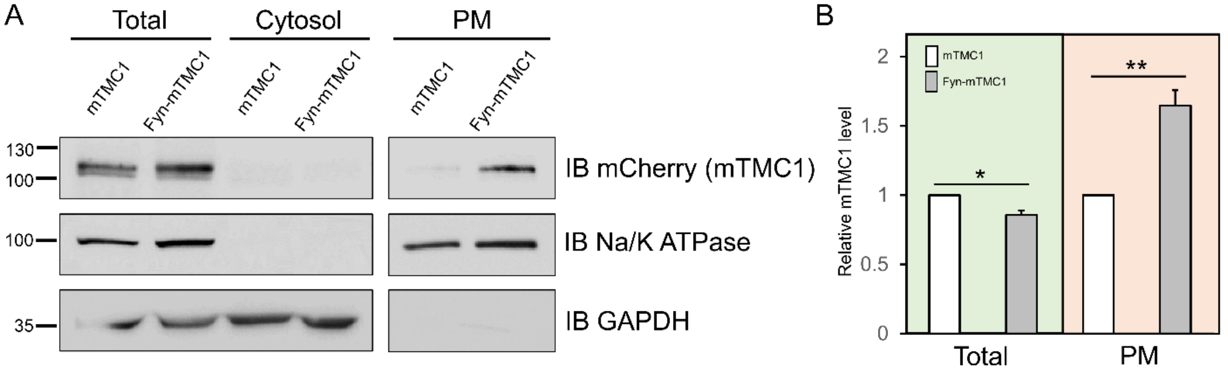

Supplementary Figure 3. Plasma membrane isolation assays confirming plasmalemmal expression of Fyn-mTMC1 in PK-HEK293T cells. (**A**) Representative western blots of total (total) and plasma membrane (PM) fraction of mTMC1-mCherry with or without Fyn tag. Na/K ATPase: loading control for membrane proteins. GAPDH: control for soluble proteins (GAPDH is absent in PM fraction, as expected). (**B**) Summary data of 3 experiments similar to that in Panel A. $**p = 0.0044$; $*p = 0.011$. Relative intensity was normalized to mTMC1-mCherry without Fyn tag.

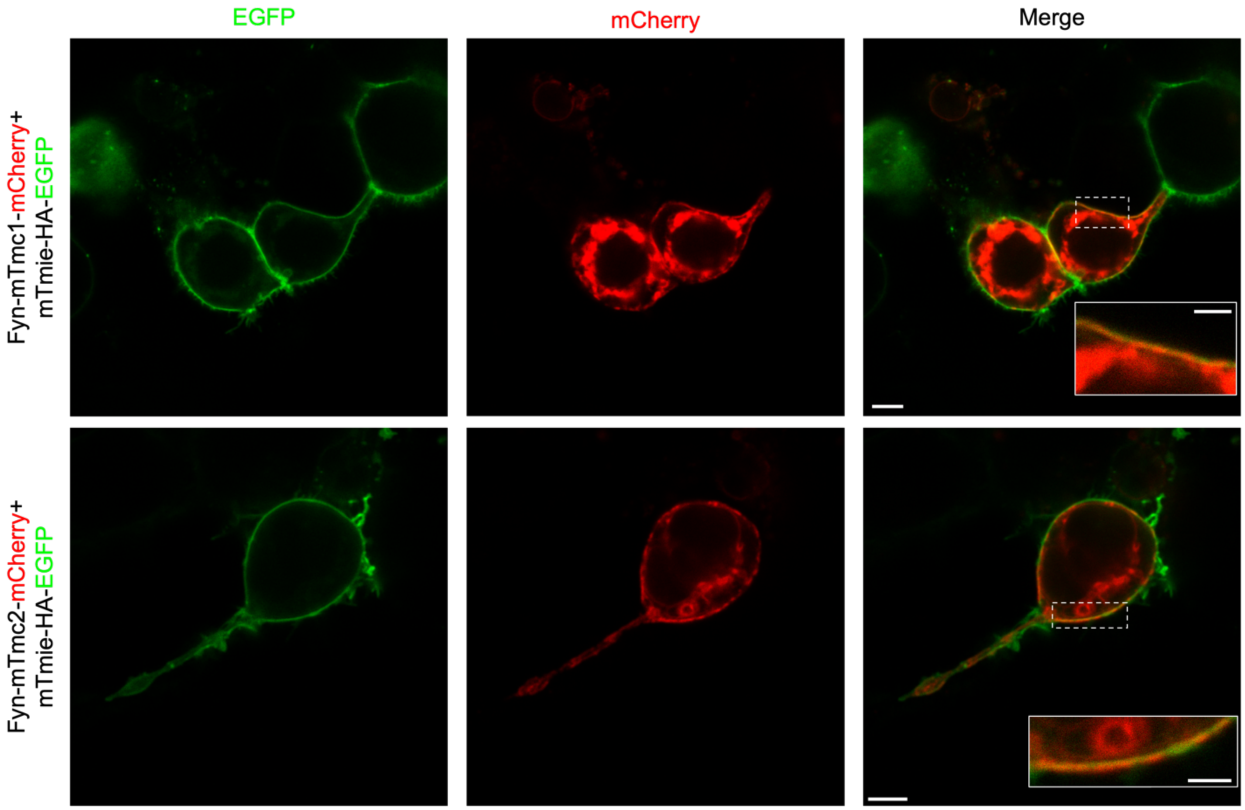

Supplementary Figure 4. Plasmalemmal colocalization of Fyn-TMC1/2-mCherry and mTMIE-EGFP. Shown are confocal images of PK-HEK293T cells expressing Fyn-mTMC1/2-mCherry (red) and mTMIE-EGFP (green). White boxes in last column: enlargement of area indicated by dotted white boxes. Scale bars: 5 µm (2 µm inside white boxes).

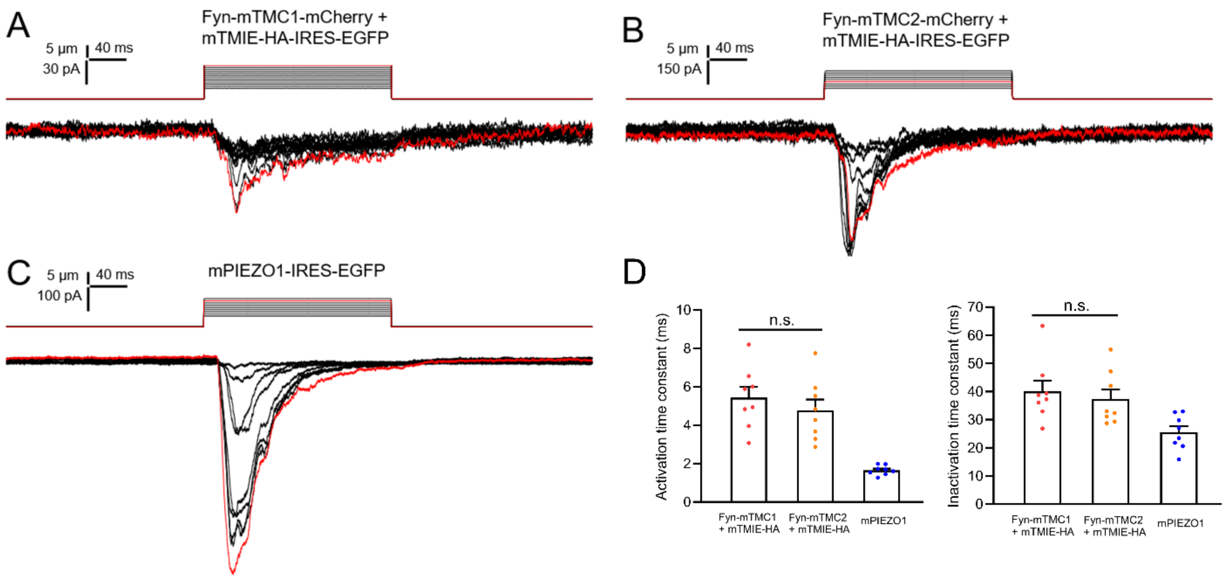

Supplementary Figure 5. Time course of activation and inactivation of MS whole-cell currents mediated by Fyn-mTMC1/2-mCherry+mTMIE. (**A-C**) Representative traces of MS whole-cell currents in PK-HEK293T cells expressing Fyn-mTMC1-mCherry+mTMIE-HA (**A**), Fyn-mTMC2-mCherry+mTMIE-HA (**B**), or mPIEZO1 as a control (**C**). Red current traces correspond to the red indentations above. (**D**) Summary data of activation and inactivation time constants of experiments in Panels A-C. Total 8 cells were analyzed in all 3 groups. n.s., not significant.

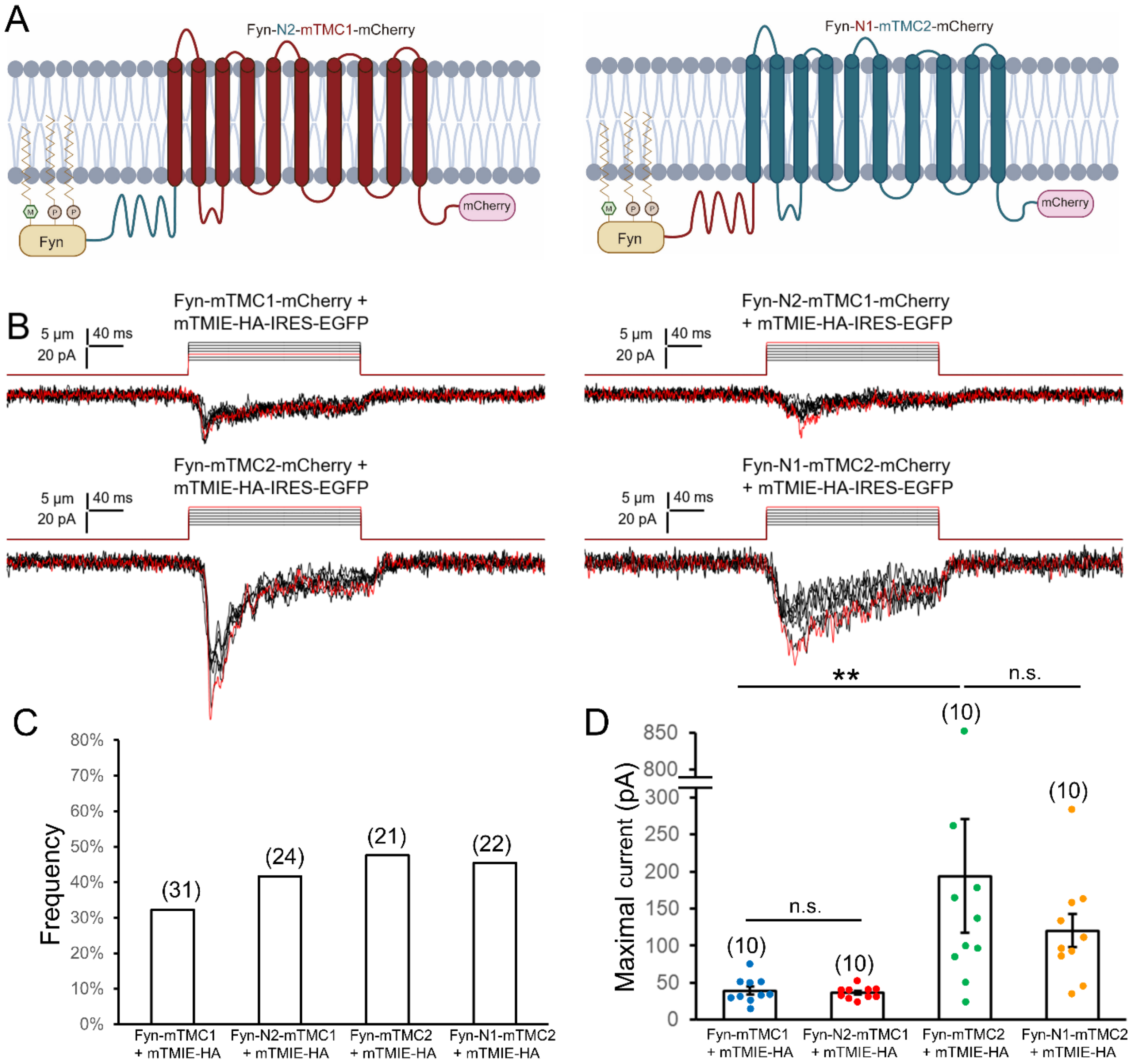

Supplementary Figure 6. Swapping N-termini between Fyn-mTMC1-mCherry and Fyn-mTMC2-mCherry does not markedly alter their MS channel activity. (**A**) Schematic of Fyn-N2(1–228 of mTMC2)-mTMC1(177–757)-mCherry and Fyn-N1(1–176 of mTMC1)-mTMC2(229–888)-mCherry. (**B**) Representative traces of MS whole-cell currents in PK-HEK293T cells expressing wild-type or chimeric Fyn-mTMC1/2-mCherry with or without mTMIE. Cells were mechanically stimulated through stepwise indentation (1 μm/step, shown above current traces). Vm = -80 mV. Red current traces correspond to the red indentations above. (**C**) Frequency of tested cells that displayed MS whole-cell currents. Number in parenthesis: number of cells tested. (**D**) Summary data of maximal MS currents in cells expressing wild-type or chimeric Fyn-mTMC1/2-mCherry. Number in parenthesis: total number of cells displaying MS current. ***p* = 0.0032; n.s., not significant. Mann-Whitney U test was used for statistical analysis.

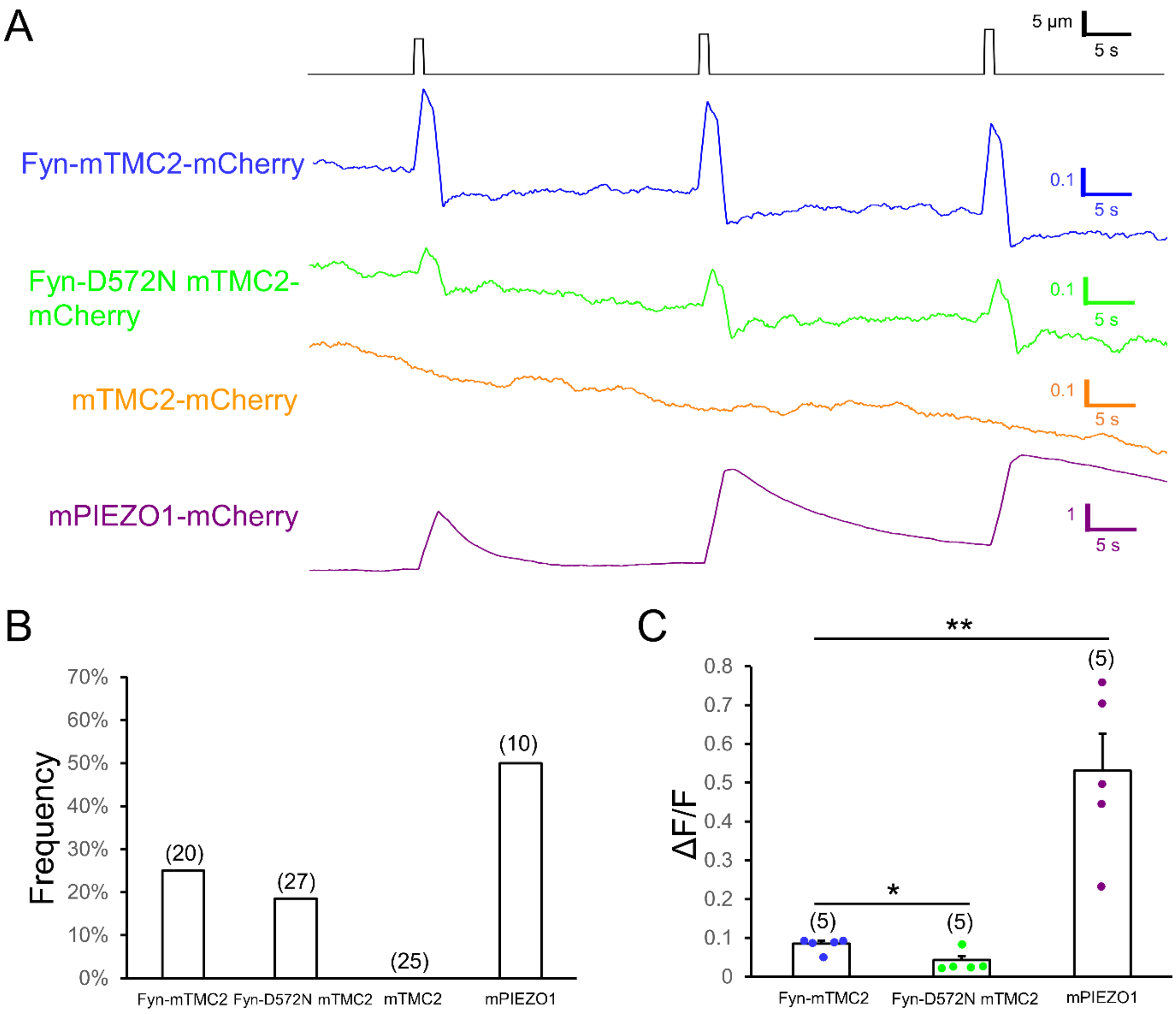

Supplementary Figure 7. Fyn-mTMC2 generates indentation-stimulated calcium influx. (**A**) Representative calcium response traces of PK-HEK293T cells transfected with Fyn-mTMC2-mCherry, Fyn-D572N mTMC2-mCherry, mTMC2-mCherry, or mPIEZO1-mCherry. Calcium indicator: Fluo-4-AM. Tested cells were visually identified based on mCherry expression and mechanically stimulated through stepwise indentation (2 μm/step, black trace on top). Fluorescence intensity is expressed in ΔF/F. (**B**) Frequency of tested cells that displayed MS calcium responses (for all groups in Panel A). Number in parenthesis: number of cells tested. (**C**) Summary data of maximal indentation-stimulated calcium response in cells that expressed Fyn-mTMC2, Fyn-D572N mTMC2, or mPIEZO1 and showed MS responses. *p = 0.0102; **p = 0.0016.

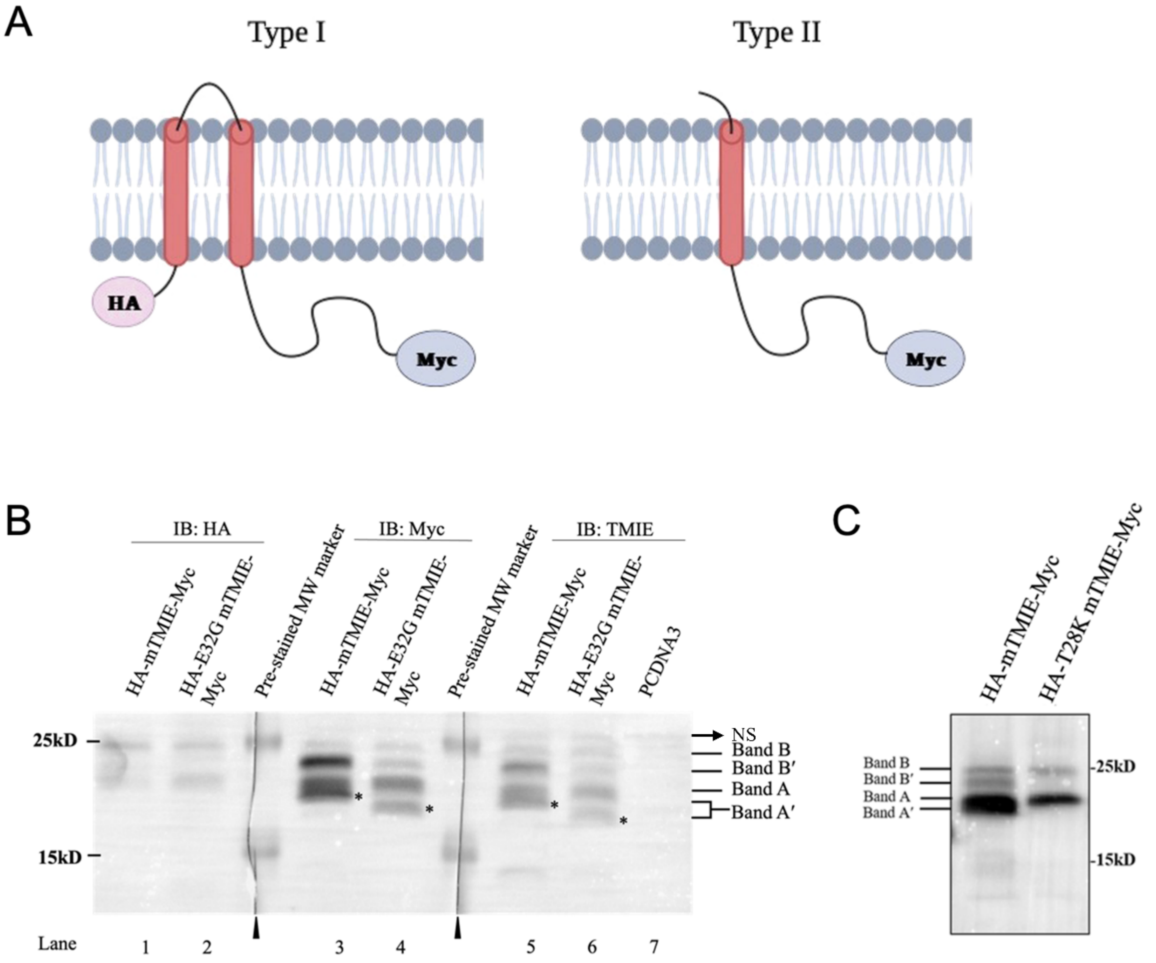

Supplementary Figure 8. Cleavage of mTMIE SP is blocked by T28K mutation. (**A**) Schematic of HA-TMIE-Myc without (left) and with (right) SP cleavage, showing their adoption of Type I and II topology, respectively. (**B**) We constructed HA-mTMIE-Myc and expressed it in HEK293T cells, and immunoblotting with anti-HA revealed two bands, of ~25 and 21 kDa (Bands B and A, respectively, lanes 1–2). Conversely, immunoblotting with anti-Myc or anti-TMIE (Cat.# HPA038298; precise epitope not reported by Sigma-Aldrich) detected two additional bands (Bands A′ and B′, lanes 3–6). These results suggest that the N-terminus is cleaved in Bands A′ and B′ and TMIE harbors a cleavable SP. Bands A′ and B′ but not A and B were observed in the Gu et al. study of hTMIE (10), suggesting more extensive SP cleavage in their study. In experiments shown in Panels B and C, 12% SDS gels were used for clear resolution of proteins ~25 kDa in size. NS, nonspecific band. (**C**) T28K mTMIE appeared as two bands with similar gel mobility as Bands A and B but not Bands A′ and B′. These results experimentally confirmed that mTMIE possesses a cleavable SP that ends before T28 and that the SP cleavage is blocked by the T28K mutation.

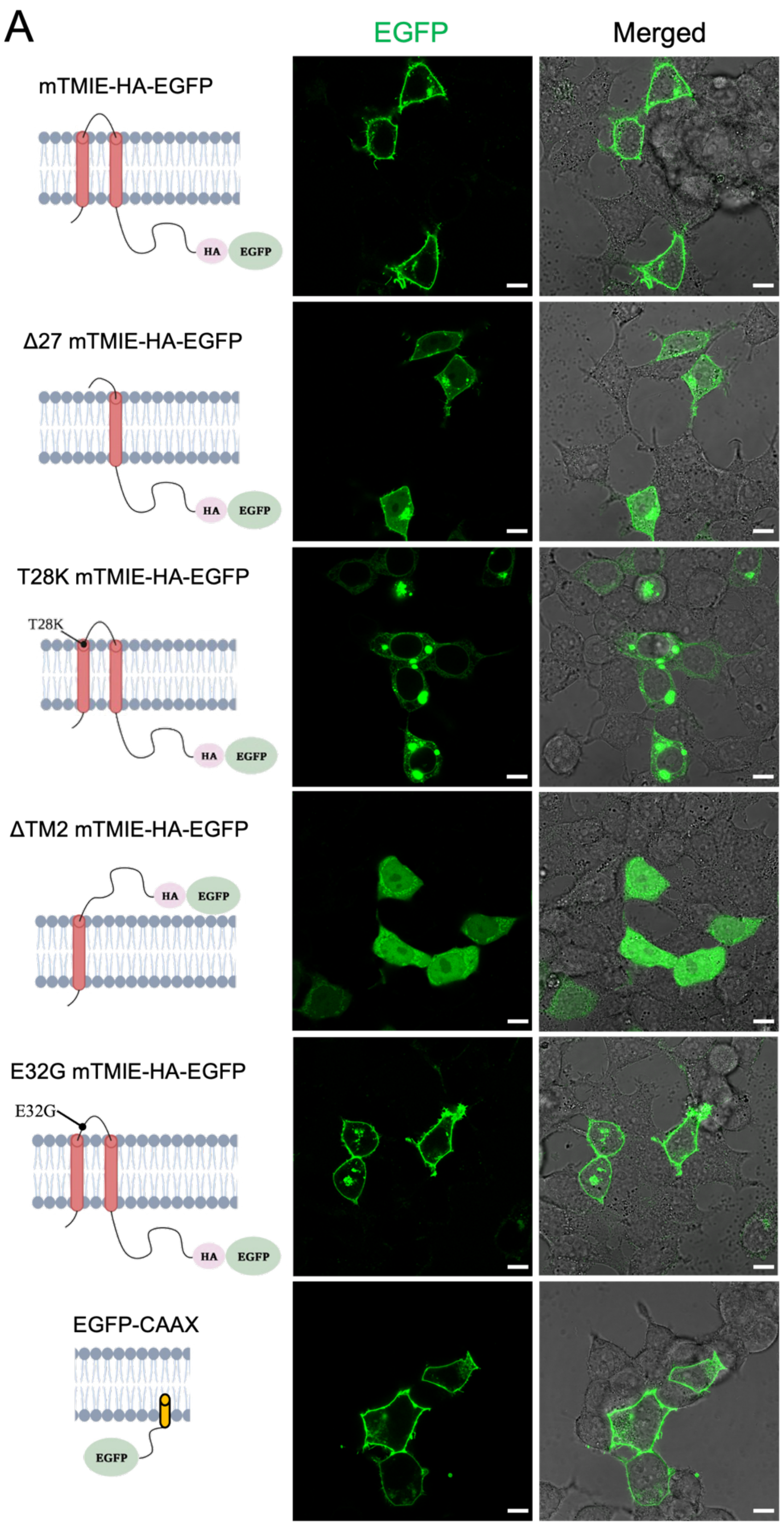
A
EGFP
Merged
mTMIE-HA-EGFP
HA
EGFP
Δ27 mTMIE-HA-EGFP
HA
EGFP
T28K mTMIE-HA-EGFP
T28K
HA
EGFP
ΔTM2 mTMIE-HA-EGFP
HA
EGFP
E32G mTMIE-HA-EGFP
E32G
HA
EGFP
EGFP-CAAX
EGFP

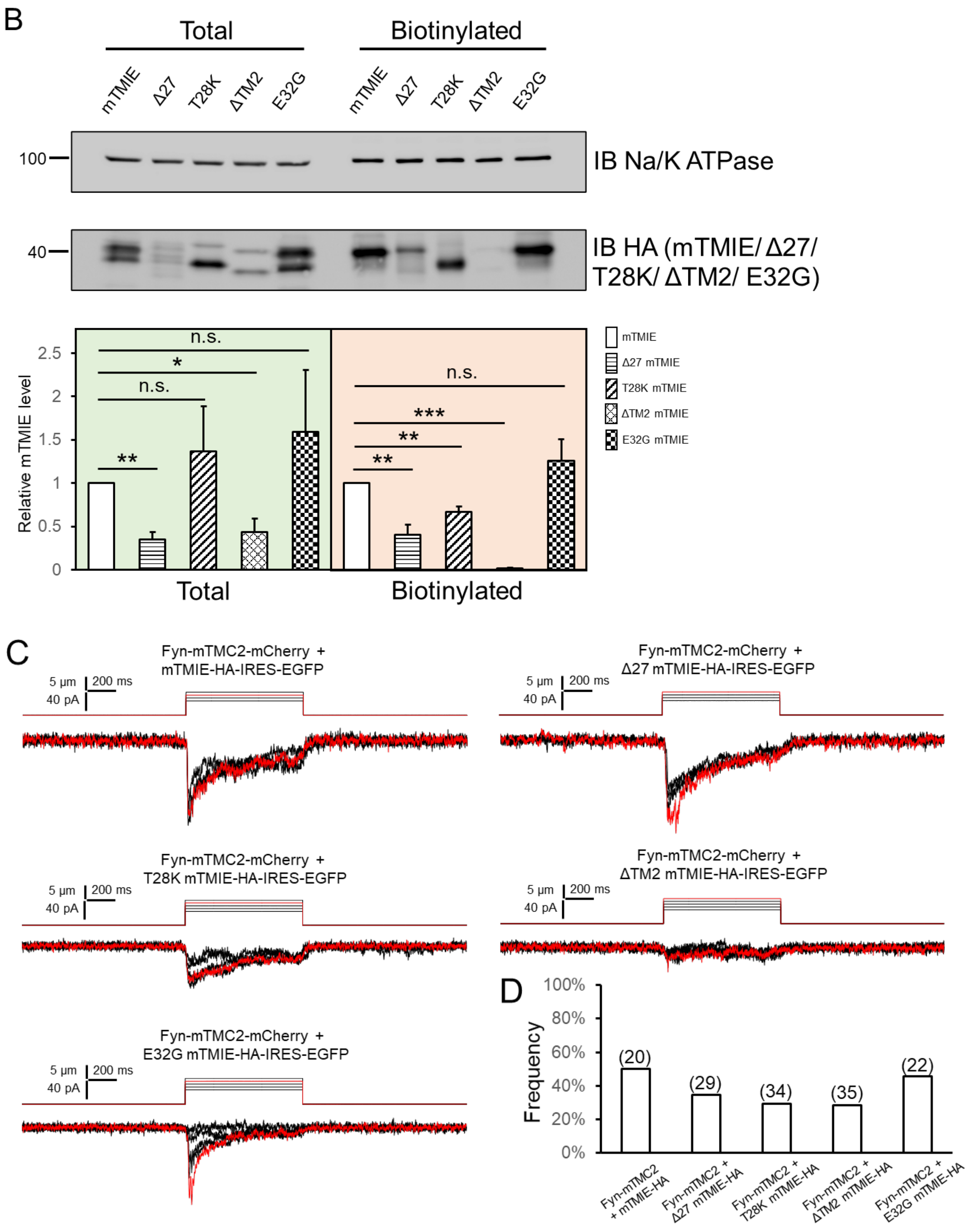
B
Total
Biotinylated
mTMIE
Δ27
T28K
ΔTM2
E32G
100
IB Na/K ATPase
40
IB HA (mTMIE/ Δ27/
T28K/ ΔTM2/ E32G)
Relative mTMIE level
0
0.5
1
1.5
2
2.5
n.s.
*
n.s.
**
***
**
**
mTMIE
Δ27 mTMIE
T28K mTMIE
ΔTM2 mTMIE
E32G mTMIE
C
Fyn-mTMC2-mCherry +
mTMIE-HA-IRES-EGFP
5 µm
200 ms
40 pA
Fyn-mTMC2-mCherry +
Δ27 mTMIE-HA-IRES-EGFP
Fyn-mTMC2-mCherry +
T28K mTMIE-HA-IRES-EGFP
Fyn-mTMC2-mCherry +
ΔTM2 mTMIE-HA-IRES-EGFP
Fyn-mTMC2-mCherry +
E32G mTMIE-HA-IRES-EGFP
D
Frequency
100%
80%
60%
40%
20%
0%
(20)
(29)
(34)
(35)
(22)
Fyn-mTMC2 + mTMIE-HA
Fyn-mTMC2 + Δ27 mTMIE-HA
Fyn-mTMC2 + T28K mTMIE-HA
Fyn-mTMC2 + ΔTM2 mTMIE-HA
Fyn-mTMC2 + E32G mTMIE-HA

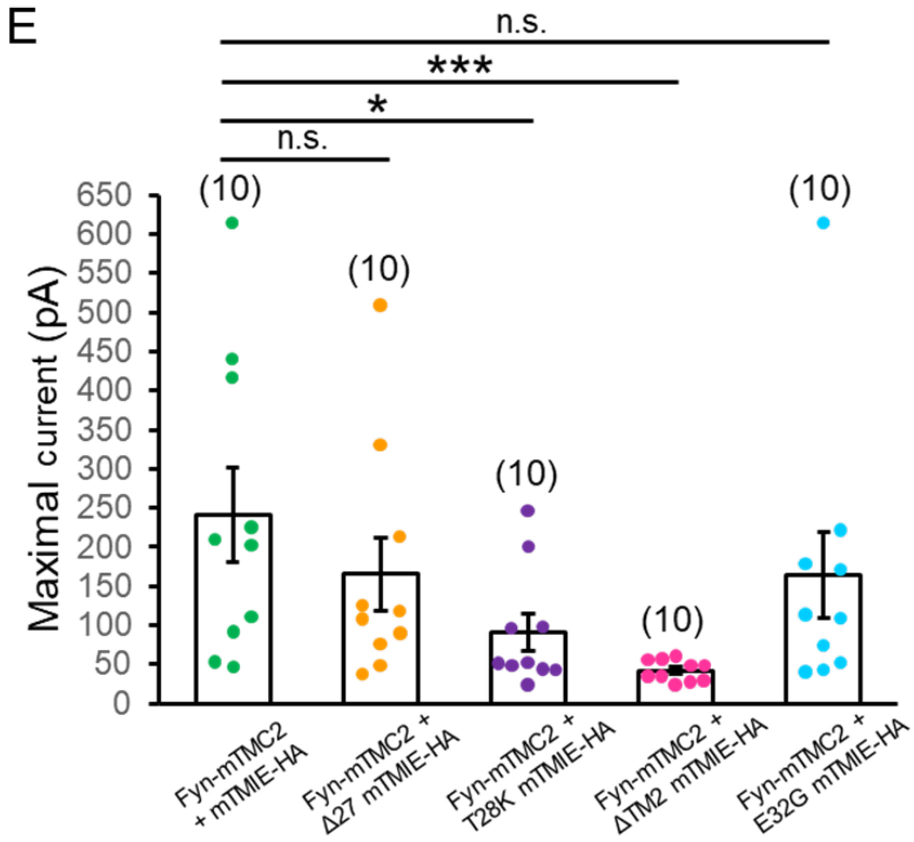

Supplementary Figure 9. Functional impact of SP of TMIE. (**A**) Schematic topology (left) and confocal fluorescence imaging (right) of wild-type and mutant mTMIE-HA-EGFP in PK-HEK293T cells. The diagrams depict the topology before SP cleavage and show that T28K mutation blocks the SP cleavage (SFig. 8). EGFP-CAAX: plasma membrane marker. Scale bars: 10 µm. (**B**) Total (total) and cell-surface (biotinylated) wild-type and mutant mTMIE-HA-EGFP. Top panel: representative western blot; bottom panel: summary data of 3 experiments similar to that in top panel. **p* = 0.021; ***p* ≤ 0.0068; ****p* = 1.2E-8; n.s., not significant. Na/K ATPase: loading control. Δ27 TMIE shows lower gel mobility relative to wild-type TMIE; the apparent irregular mobility of Δ27 TMIE was also reported previously (11) and probably results from a reduction in the overall hydrophobicity of the protein due to the N-terminal deletion (12). (**C**) Representative traces of MS whole-cell currents in PK-HEK293T cells expressing Fyn-mTMC2-mCherry with wild-type or mutant mTMIE-HA constructs. Cells were mechanically stimulated through stepwise indentation (1 µm/step, shown above current traces). Vm = -80 mV. Red current traces correspond to the red indentations above. (**D**) Frequency of tested cells that displayed MS whole-cell currents. Number in parenthesis: number of cells tested. (**E**) Summary data of maximal MS current in cells expressing Fyn-mTMC2-mCherry with wild-type or mutant mTMIE-HA constructs. Number in parenthesis: total number of cells displaying MS current. **p* = 0.031; ****p* = 0.0007253; n.s., not significant. Mann-Whitney U test was used for statistical analysis.

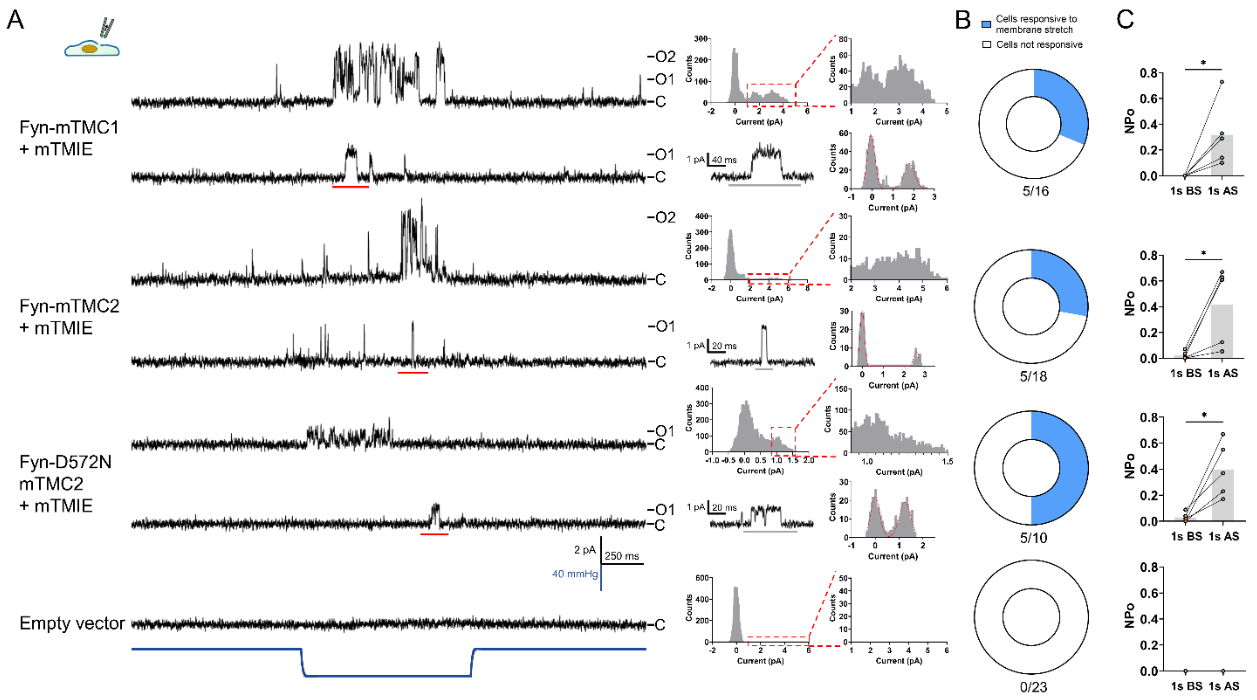

Supplementary Figure 10. Single-channel study of Fyn-mTMC1/2-mCherry in PK-HEK293T cells. (**A**): Representative MS single-channel traces in excised, inside-out membrane patches of PK-HEK293T cells co-expressing Fyn-mTMC1-mCherry, or Fyn-mTMC2-mCherry, or Fyn-D572N-mTMC2-mCherry and mTMIE. Vm = +100 mV. C: closed state of channel; O1 and O2: open state of one or two channels; blue line: negative pressure of 40 mmHg applied to membrane patches. In Panel A, the bath and pipette solution contained (in mM) 150 NaCl and 10 HEPES (pH 7.3 with NaOH). For the upper current trace in each condition, the all-points amplitude histograms during application of negative pressure are shown at the right. The low current trace emphasizes events of the unitary conductance and the expansion of its red-underlined segment is present at the left, in which the gray-underlined segment was used for amplitude histogram. (**B**) Donut charts of proportion of tested cells that displayed MS single channels in membrane patches. 10–23 independent cells were tested in different experimental groups. (**C**) Summary data of NP_o 1 sec before and after negative pressure in 5 independent experiments similar to Panel A. 5, 20, 40, and 60 mmHg negative pressure were applied to each membrane patch and maximal mechanical response in each membrane patch was used for analysis. *$p \leq 0.0464$. A low-pass filter at 0.5 kHz was applied in all experiments presented in SFig. 10.

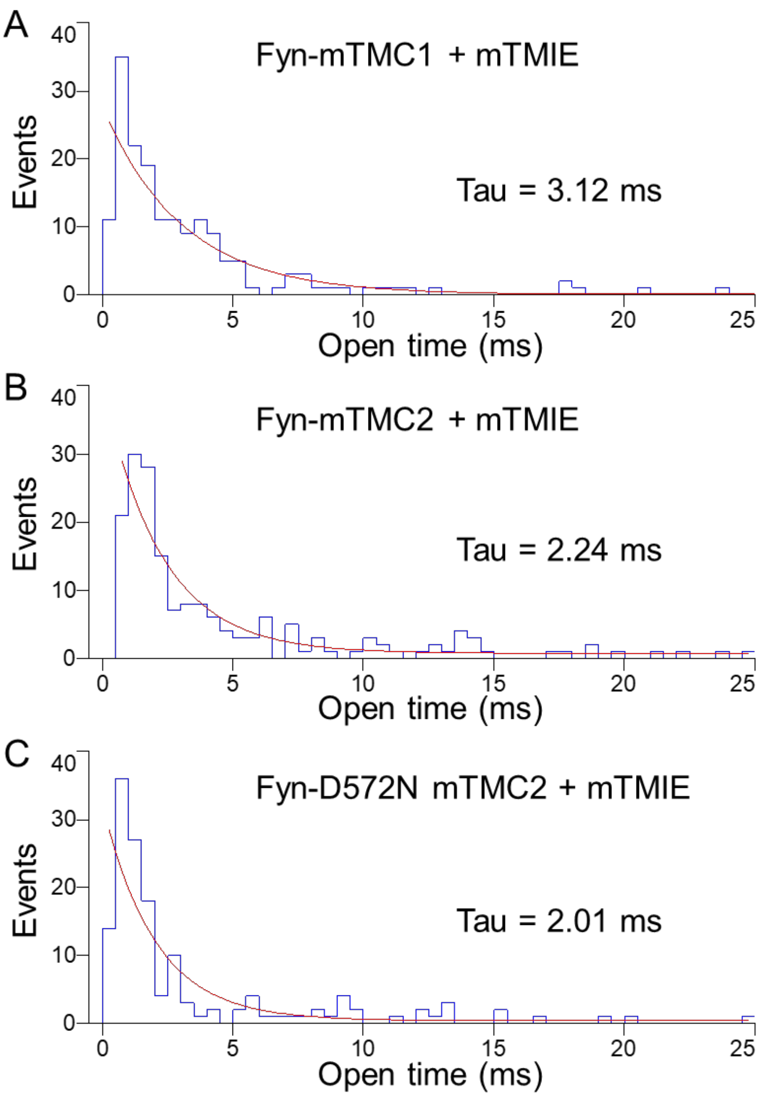

Supplementary Figure 11. Histograms of open dwell time of Fyn-mTMC1/2+mTMIE. Open dwell times were extracted from single-channel analyses shown in Fig. 5 and SFig. 10. Red curves represent single exponential fits with a time constant Tau for Fyn-mTMC1-mCherry + mTMIE (**A**, 180 events from 24 cells), Fyn-mTMC2-mCherry + mTMIE (**B**, 185 events from 31 cells), and Fyn-D572N-mTMC2-mCherry + mTMIE (**C**, 155 events from 23 cells). Note that events longer than 25 ms are relatively few and were excluded from the analysis.

SI Appendix References

1. X. J. Yu *et al.*, Deafness mutation D572N of TMC1 destabilizes TMC1 expression by disrupting LHFPL5 binding. *Proceedings of the National Academy of Sciences of the United States of America* **117**, 29894-29903 (2020).
2. Y. Sun, F. Wu, F. Q. Sun, P. B. Huang, Adenosine promotes IL-6 release in airway epithelia. *Journal of Immunology* **180**, 4173-4181 (2008).
3. W. Hu *et al.*, The complex of TRIP-Br1 and XIAP ubiquitinates and degrades multiple adenylyl cyclase isoforms. *Elife* **6** (2017).
4. Y. Duan *et al.*, Keratin K18 increases cystic fibrosis transmembrane conductance regulator (CFTR) surface expression by binding to its C-terminal hydrophobic patch. *J Biol Chem* **287**, 40547-40559 (2012).
5. M. Moroni, M. R. Servin-Vences, R. Fleischer, O. Sanchez-Carranza, G. R. Lewin, Voltage gating of mechanosensitive PIEZO channels. *Nat Commun* **9**, 1096 (2018).
6. S. Liu *et al.*, TMC1 is an essential component of a leak channel that modulates tonotopy and excitability of auditory hair cells in mice. *Elife* **8** (2019).
7. K. X. Kim, R. Fettiplace, Developmental changes in the cochlear hair cell mechanotransducer channel and their regulation by transmembrane channel-like proteins. *Journal of General Physiology* **141**, 141-148 (2013).
8. A. H. Truesdell, Activity Coefficients of Aqueous Sodium Chloride from 15degrees to 50degreesC Measured with a Glass Electrode. *Science* **161**, 884-886 (1968).
9. J. A. Rard, S. L. Clegg, Critical Evaluation of the Thermodynamic Properties of Aqueous Calcium Chloride. 1. Osmotic and Activity Coefficients of 0−10.77 mol·kg-1 Aqueous Calcium Chloride Solutions at 298.15 K and Correlation with Extended Pitzer Ion-Interaction Models. *Journal of Chemical & Engineering Data* **42**, 819-849 (1997).
10. S. Gu *et al.*, Hair cell alpha9alpha10 nicotinic acetylcholine receptor functional expression regulated by ligand binding and deafness gene products. *Proc Natl Acad Sci U S A* **117**, 24534-24544 (2020).
11. C. L. Cunningham *et al.*, TMIE Defines Pore and Gating Properties of the Mechanotransduction Channel of Mammalian Cochlear Hair Cells. *Neuron* **107**, 126-143 e128 (2020).
12. A. Shirai *et al.*, Global analysis of gel mobility of proteins and its use in target identification. *J Biol Chem* **283**, 10745-10752 (2008).
